# Hawaiian *Paratachys* Casey (Coleoptera, Carabidae): small beetles of sodden summits, stony streams, and stygian voids

**DOI:** 10.3897/zookeys.1044.59674

**Published:** 2021-06-16

**Authors:** James K. Liebherr

**Affiliations:** 1 Department of Entomology, John H. and Anna B. Comstock Hall, 129 Garden Ave., Cornell University, Ithaca, NY 14853-2601, USA Cornell University Ithaca United States of America

**Keywords:** Brachyptery, flight-wing dimorphism, ocular polymorphism, troglobite

## Abstract

Five Hawaiian species of *Paratachys* Casey are revised, including four newly described: *Paratachys terryli* from Kauai; *P. perkinsi* from Moloka‘i; *P. haleakalae* from Maui; and *P. aaa* from Hawai‘i Island. A lectotype is designated for the fifth Hawaiian species currently combined with *Paratachys*, *Tachys arcanicola* Blackburn, 1878 of Oahu. Hawaiian *Paratachys* spp. known from more than one specimen exhibit some degree of ocular polymorphism, that variation being extreme in *P. terryli* where individuals range in ocular development from macrophthalmic with broadly convex eyes to microphthalmic with small, flat eyes. All Hawaiian *Paratachys* species comprise individuals with vestigial wings, with the exception of *P. terryli*, where a single macropterous, macrophthalmic female complements the other 18 brachypterous specimens. Based on a transformation series of characters from the male aedeagus, the biogeographic history of Hawaiian *Paratachys* is consistent with progressive colonization of the Hawaiian Island chain. Three of the species do not appear to represent species of conservation concern, with *P. terryli* and *P. haleakalae* known from terrestrial deep soil, litter, and streamside microhabitats in montane wet rain forest, and the troglobitic *P. aaa* occupying the dark zone of numerous, recently developed lava tube caves within the Mauna Loa and Kilauea volcanic massifs. The conservation status of the other two species is much more dire, with *P. arcanicola* of O‘ahu not seen in nature since the early 20^th^ Century, and *P. perkinsi* known only from a single specimen fortuitously collected in 1894 near sea level on Moloka‘i.

*“Tachys ...*

*The species of this genus are of comparatively little interest...*

*They are most obscure, minute insects of a kind*

*that occurs in various other parts of the world*

*(Sharp 1903: 287).”*

## Introduction

Terry Erwin conducted doctoral dissertation research with Professor George E. Ball at the University of Alberta on arguably the most charismatic group of carabid beetles; the bombardier beetles (Erwin 1970). These beetles have been studied subsequently by workers who have elucidated their explosive, crepitating defensive behavior (Eisner and Aneshansley 1999; Armitage 2004; Arndt et al. 2015; Di Giulio et al. 2015; Attygalle et al. 2020), aposematism associated with that defensive behavior (Brandmayr et al. 2006; Schaller et al. 2018), as well as their parasitic larval way of life (Saska and Honek 2004, 2008). However, Terry changed course and in 1971 published the first of many papers on obscure, minute trechite carabid beetles, describing two new fossil species and a new genus, *Tarsitachys* Erwin. A year later he described the Austral genera *Bembidarenas* Erwin of South America and *Tasmanitachoides* Erwin of Australia (Erwin 1972). He continued to focus considerable efforts on revisions of various trechite taxa, including *Xystosomus* Schaum (Erwin 1973), *Pericompsus* LeConte (Erwin 1974a), *Costitachys* Erwin and *Meotachys* Erwin (Erwin 1974b, 1991; Erwin and Kavanaugh 1999, 2007), *Tachyta* Kirby (Erwin 1975), and the subtribe Xystosomina Erwin, proposing in that work four new genera and 40 new species (Erwin 1994). The most recent review of New World Tachyina sensu Boyd and Erwin (2016) lists 13 Erwin-described generic-level taxa, doubling the number of previously proposed generic names for the fauna. Terry Erwin left us with many legacies, among them a profoundly better understanding of the obscure, minute beetles allied with *Tachys* Stephens.

This contribution presents a taxonomic revision of the native Hawaiian carabid beetles assignable to the genus *Paratachys* Casey in the sense of Boyd and Erwin (2016). This taxon has been long recognized as distinct; e.g., Hayward (1900) treating members as the *proximus* species group. Erwin (1974b) recognized *Paratachys* as a generically distinct taxon, synonymizing *Eotachys* Jeannel with Casey’s name. At that time, Erwin compounded his previous experience working with the carabid beetle collections of The Natural History Museum, London and the Muséum national d’Histoire naturelle, Paris, with his position as Curator of Coleoptera at the National Museum of Natural History, repository of the Thomas Lincoln Casey Collection of Coleoptera, to gain a worldwide view of the diverse lineages that comprise the presently recognized tribe Tachyini (Maddison et al. 2019).

There is a single previously described Hawaiian species, *P. arcanicola* (Blackburn) of Oahu, currently assigned to *Paratachys* Casey (Erwin 1974b). During field surveys supporting revisions of native Hawaiian Carabidae, i.e., beetles of the tribes Platynini (Liebherr and Zimmerman 2000) and Moriomorphini (Liebherr 2015), plus the genus *Bembidion* Latreille (Liebherr 2008a), specimens representing two undescribed *Paratachys* were discovered in wet rain forest habitats, one each on the islands of Kauai and Maui. Careful examination of museum specimens collected by Rev. Thomas Blackburn and Dr. R.C.L. Perkins, deposited in The Natural History Museum, London, demonstrated that a third undescribed species from lowland Moloka‘i was misidentified by Britton (1948) as a specimen of *P. arcanicola*. And fourthly, an undescribed troglobitic species, previously reported as *Tachys arcanicola* (Howarth 1973: fig. 2), has been repeatedly collected in the dark zone of lava tube caves derived from recent eruptive episodes of Mauna Loa and Kilauea volcanoes, Hawai‘i Island. Besides validating these new species and providing an identification key, infraspecific variation in eye and wing development is presented. For those species represented by multiple specimens, Hawaiian *Paratachys* are shown to exhibit variation in eye development. Moreover, the new species from Kauai exhibits flight-wing dimorphism, with one individual out of the 19 known bearing fully developed, well-veined wings that are of a configuration consistent with that observed for beetles of another flight capable Hawaiian tachyine, *Tachys oahuensis* Blackburn. Though these beetles may be minute and obscure, they are immensely interesting, and this contribution honors Terry Erwin’s long-term efforts elucidating the biodiversity of trechite carabid beetles.

## Materials and methods

This revision is based on 90 specimens which are deposited in the following institutions: Bernice P. Bishop Museum, Honolulu, HI (**BPBM**); California Academy of Sciences, San Francisco, CA (**CAS**); Luca Toledano private collection, Verona, Italy (**CTVR**); Cornell University Insect Collection (**CUIC**); Muséum national d’Histoire naturelle, Paris (**MNHN**); The Natural History Museum, London (**NHMUK**); National Museum of Natural History, Washington, D.C. (**NMNH**); Oregon State Arthropod Collection, Corvallis, OR (**OSAC**); University of Hawaii Insect Museum, Manoa, HI (**UHIM**).

Beetles were collected by aspirating or hand-picking specimens from streamside habitats and forest-floor litter siftate spread on a beating sheet. Bulk samples of siftate from approximately 2–3 m^2^ of forest floor were also transported to a field lab and cooked off in a double-boiler, with the near-boiling water drying the litter and concentrating the microarthropods in the center of the inner pot. Field specimens of the Kauai and Maui species were killed in potassium cyanide charged glass kill jars (1991) or ethyl acetate charged plastic 50 ml centrifuge tubes (2005), then transferred to 70% ethanol 24 hours later. Hawai‘i Island cave beetles were hand collected live and then transferred to 70% ethanol after processing in a field lab (C.A.M. Slay, pers. comm.).

External characters were assessed using dry, pointed specimens examined under halogen light sources. Various ratios were used to describe beetle anatomy, including: ocular ratio (**OR**), maximum head width across the compound eyes divided by the minimum frons width between the eyes; **MPW/BPW**, maximum pronotal width divided by the pronotal width measured between the pronotal hind angles; **MPW/PL**, maximum pronotal width divided by the pronotal length measured along the midline; **HuW/MEW**, distance between the juncture of the elytral basal groove and the lateral marginal depression (the humerus) divided by maximum elytral width; and **EL/MEW**, elytral length measured from the front of the elevated triangular portion of the scutellum to the apex of the longer elytron divided by MEW. Body size is presented as the standardized body length, i.e., the sum of the distances from the apical margin of the labrum to the cervical ridge at the back of the vertex, **PL**, and **EL**, the latter two measurements as described above.

Elytral setation and striation are presented using the system of Erwin (1974a). In this system, upraised convex elytral intervals are bordered by depressed interneurs. Erwin presented a generalized scheme of elytral setation that hypothesized a groundplan elytral setation in the Tachyini (Maddison et al. 2019). Such a groundplan permits estimation of generalized and derived presence and absence for various setae, thereby allowing setational characters to be viewed in a phylogenetic context. Cuticular microsculpture is described using the terminology of Lindroth (1974). Male and female genitalic preparations were made by clearing dissections in 10% KOH and then neutralizing the dissected parts in dilute acetic acid. Male structures were dehydrated in an alcohol series, and then stored temporarily in clove oil for viewing microscopically. Female reproductive tracts and gonocoxae were stained in Chlorazol Black (Eastman-Kodak, Rochester, NY) suspended in methyl cellosolve, and transferred to glycerin for viewing. Terminology used for the female structures follows Liebherr and Will (1998). Lengths of the female spermathecal ducts were measured by drawing the convoluted ducts to scale from the microscope slide preparation, and then using a mapping opisometer (Vintage American Map Co., Inc., New York, NY) to calculate the duct length. All genitalic structures were ultimately stored in glycerin within polyethylene genitalia vials attached to the specimen pins.

## Taxonomic treatment

### Paratachys

Taxon classificationAnimaliaColeopteraCarabidae

Casey, 1918

85256569-CEBF-5F0D-9F70-0AE77E1B7169

Paratachys Casey, 1918: 174 (type species Paratachys austinicus Casey by original designation); Erwin 1974b: 128; Boyd and Erwin 2016: 95, 110.
Eotachys Jeannel, 1941: 426 (type species Tachys bistriatus Duftschmid by original designation). Synonymy by Erwin (1971: 236).
Macrotachys Kult, 1961: 2 [junior homonym of Macrotachys Uéno, 1953: 42] (type species Bembidium fulvicolle Dejean by original designation). Synonymy under Eotachys by Lindroth (1966: 431).

#### Diagnosis.

**Figure 1.**
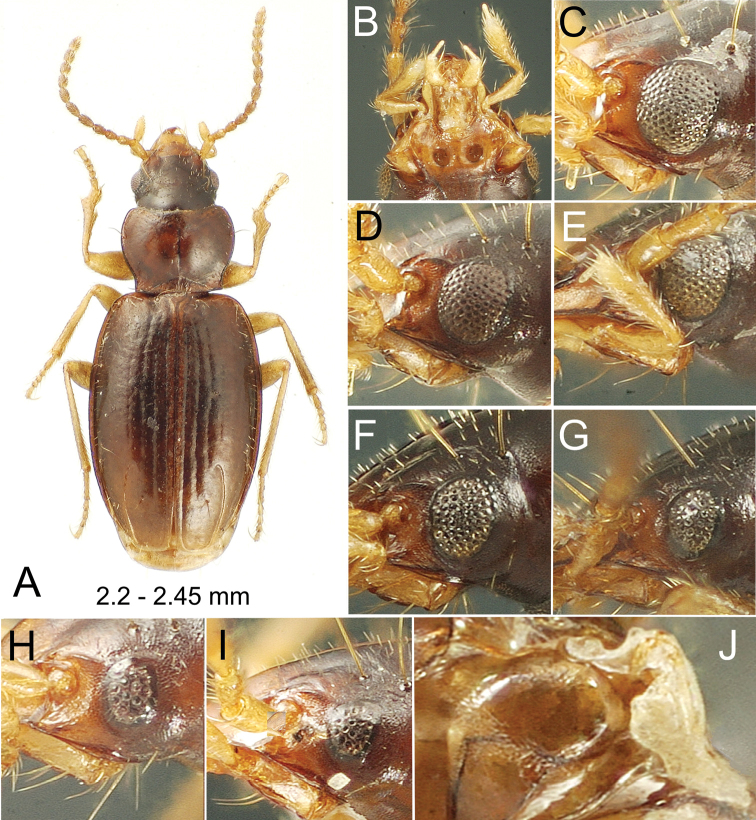
*Paratachys terryli***A–I** Kauai: Namolokama Mtn. **A, B** macrophthalmic, brachypterous female; 23-v-2005 lot 01 **A** dorsal habitus view **B** mentum and associated mouthparts, ventral view **C–I** compound eye, lateral view **C** macropterous female; 23-v-2005 lot 1 **D** brachypterous male; 21-v-2005 lot 8 **E** brachypterous male; 23-v-2005 lot 1 **F** brachypterous female; 23-v-2005 lot 1 **G** brachypterous female; 23-v-2005 lot 1 **H** brachypterous female; 23-v-2005 lot 1 **I** brachypterous female; 21-v-2005 lot 1 **J** Kauai: Upper Kawaikoi Stream at Alakai Swamp Trail, 16-v-1971; right metathoracic flight wing of male, vestigial morph (elytron removed); 14-v-1991 stop 91-26B.

These beetles can be diagnosed by: deep paramedial pits of the mentum (Figs 1B, 8B, 9B); head with two supraorbital setae each side (Figs 1C, D, 8C, D, 9D, F); pronotum moderately cordate to nearly quadrate, MPW/BPW = 1.17–1.34; anterior terminus of elytral apical recurrent groove (ARG) slightly to distinctly hooked and situated laterad position of interneur 4, ARG laterally encompassing the fourth discal seta Ed5–6 (Figs 1A, 6, 8A, 9A); elytral setation including ombilicate setae in an anterior series Eo1–4, a posterior series Eo5–8 associated with posterior sulcus, and Eo9 just mesad ARG, plus dorsal setae Ed1, Ed5–6, Ed7, and Ed8; interneur 8 with posterior sulcus in apical half of elytron, the anterior portion of sulcus directed away from the elytral lateral margin and encompassing setae Eo5–6, and setae Eo7 and Eo8 situated toward the elytral apex between i8 and the apical recurrent groove; and protibia expanded laterally at apical 1/4 of length (Fig. 1A), the associated latero-apical notch lined basally with a transverse row of closely spaced setae (e.g., Maddison et al. 2019: fig. 7A).

For Hawaiian taxa the position of the dorsal seta within the arcuate ARG differs from that in Neotropical *Paratachys* (Boyd and Erwin 2016) by being situated near the middle of the arc (Figs 1A, 8A, 9A), not further forward near the apical hooked terminus of the groove (e.g., Boyd and Erwin 2016: fig. 1A, C). This suggests that the seta within the arc may represent Ed6 in Hawaiian *Paratachys*, vs. Ed5 in Neotropical taxa (Boyd and Erwin 2016: fig. 1A). An anterior placement of the seta mesad the hooked terminus of the ARG is also observed in Asian *Paratachys* (Andrewes 1925: plate 3; Tanaka 1956: fig. 1; Tanaka 1960: fig. 1; Habu 1977: fig. 1; Baehr 2017: figs 29–34), the Palaearctic *P. bistriatus* (Duftschmid) (Jeannel 1941: fig. 168A) as well as numerous other Palaearctic species (Coulon 2004: figs 2–4), various Madagascan species (Jeannel 1946: fig. 154), Canary Island species (Machado 1992: fig. 73C, D), and Nearctic taxa such as *P. proximus* (Say) and *P. scitulus* (LeConte) (Lindroth 1966: figs 209f, 222).

Finally, all Hawaiian species have the cuticular surfaces of head, prothorax, elytra, meso- and metathoracic sternites, abdominal ventrites, and femora covered with a pelage of fine microsetae (Figs 1C–I, 8C, D, 9C–F). These microsetae occur in line with the elytral interneurs, and on the compound eyes are set along the ommatidial margins (Figs 1B, 8B, 9B). Again, this configuration differs from that of Nearctic taxa such as *P. austinicus* Casey, *P. proximus*, and *P. scitulus* (CUIC), in which only the meso- and metathoracic femora bear such a pelage and all other body surfaces are glabrous excepting macrosetae. It should be noted that some *Tachys* spp. also have the ventral surface of the meso- and metathoracic femora covered with a sparse pelage; e.g., *T. litoralis* Casey, *T. mordax* LeConte, and *T. vittiger* LeConte. *Setitachys macrops*Baehr (2016) of northern Australia also bears a setose pelage over the body, but this species differs dramatically from the Hawaiian *Paratachys* in mandibular length, pronotal shape, elytral striation, and aedeagal configuration, suggesting an independent origin for the pelage in the two lineages. Species of the *Lymnastis* Motschulsky-*Micratopus* Casey lineage (Maddison et al. 2019) are also characterized by such a full-body pelage (Baehr 2016).

#### Identification and generic placement.

Boyd and Erwin’s (2016) key to New World Tachyina supports generic assignment of Hawaiian *Paratachys* through use of the characters that diagnose the genus worldwide. Andrewes’ (1925) key to Oriental *Tachys* Dejean sensu lato is based on a similar though simplified set of characters that assigns Hawaiian *Paratachys* species to his “*triangularis*” group. Finally, Britton (1948) could be used firstly to generically determine Hawaiian *Paratachys* as *Tachys*, and then as the only previously described species, *Paratachys arcanicola* (Blackburn) (Britton 1948: 238), although Britton’s keys do not utilize diagnostic characters (Boyd and Erwin 2016) that underlie taxonomic definition of *Paratachys*.

Coulon (2004) distinguished European species of *Eotachys* Jeannel from those of *Paratachys* based on the complete eighth elytral interneur of the Nearctic *P. austinicus* Casey, type species of *Paratachys*, vs. an interrupted eighth interneur in the European *Tachys bistriatus* Duftschmid, type species of *Eotachys*. However, as the European *Tachys bistriatus* is nested within a clade of New World *Paratachys* spp., with the Hawaiian *Paratachys terryli* (reported as *Paratachys* sp. “U.S.A.: Hawaii”) placed as their respective adelphotaxon in a comprehensive molecular phylogenetic analysis (Maddison et al. 2019, fig. 12), the synonymy of *Eotachys* under *Paratachys* reported first by Erwin (1971) is corroborated, and herein accepted.

### Key to the adults of Hawaiian *Paratachys* Casey

**Table:** 

1	Body size larger, standardized body length 2.2–2.5 mm; apical recurrent groove (ARG) deep, elytral surface broadly depressed mesad groove; elytral interneurs 1–3 evident near midlength, 1 and 2 deeply impressed, 3 traceable to evident near dorsal elytral seta, elytral intervals 1–3 equally convex	**2**
–	Body size smaller, standardized body length 1.8–2.2 mm; ARG moderately deep, elytral surface convex immediately mesad groove; elytral interneurs 1 and 2 evident near midlength, interneur 1 (i1) impressed with sutural interval broadly elevated on each elytron resulting in a callous-like suture, i2 broadly shallow, i3 obsolete near dorsal elytral seta	**4**
2	Pronotum more transverse, MPW/PL = 1.35–1.41; lateral marginal depression narrowly reflexed (Figs 1A, 6B); pronotal lateral margin convex, margin distinctly sinuate anterad hind angle	**3**
–	Pronotum narrower, MPW/PL = 1.29–1.36; lateral marginal depression very narrow, obsolete, margin carinate (Fig. 8A); pronotal lateral margin less convex, margin less distinctly sinuate anterad hind angle; East Maui I	***Paratachys haleakalae* sp. nov.**
3	Elytral marginal groove curved to join basal groove on humerus (Fig. 1A); elytra narrower basally, elytra suboviform, HuW/MEW = 0.58; Kauai I	***Paratachys terryli* sp. nov.**
–	Elytral marginal groove-basal groove juncture angulate (Fig. 6B); elytral broader basally, elytra subquadrate, HuW/MEW = 0.64; Moloka‘i I	***Paratachys perkinsi* sp. nov.**
4	Eyes variable, small but outer surface always more convex than head curvature of gena (Fig. 6A), from 5–7 ommatidia crossed by horizontal line bisecting eye, from 6–9 ommatidia crossed by vertical line; O‘ahu I	***Paratachys arcanicola* (Blackburn)**
–	Beetles microphthalmic, outer surface of eye nearly flat, not extended beyond curvature of gena (Fig. 9A), ommatidia difficult to discern because of microsculpture on cuticle covering eye, from 3–5 ommatidia crossed by horizontal line bisecting eye, from 4–6 ommatidia crossed by vertical line (Fig. 9C–F); Hawai‘i I	***Paratachys aaa* sp. nov.**

### Paratachys terryli sp. nov.

Taxon classificationAnimaliaColeopteraCarabidae

29E43575-8D6D-5438-AF09-35206D2E7210

http://zoobank.org/9B7B5D1A-8243-4BBE-A95F-DE1477915432

**Figure 2.**
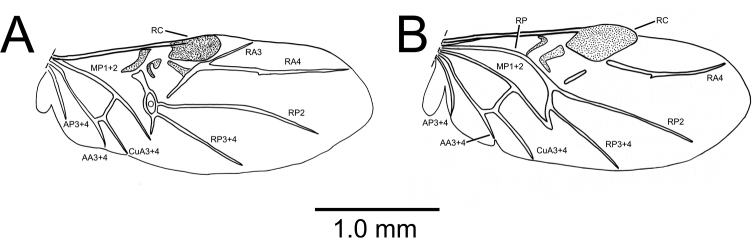
Right metathoracic flight wing, dorsal view **A***Paratachys terryli*, macrophthalmic female; Kauai: Namolokama Mtn., 23-v-2005 (CUIC) **B***Tachys oahuensis* Blackburn, female; Oahu: Honolulu, Public Health Dept. light trap, x-1965, J. W. Beardsley (BPBM). Wing vein terminology follows Kukalová-Peck and Lawrence (1993) except for the oblongum cell of Forbes (1922). From front to back of wing, veins, and cells relevant to the text include the Radial Cell (RC). Radius Anterior (RA), Radius Posterior (RP), Oblongum cell (O), Media Posterior (MP), Cubitus Anterior (CuA), Anal Anterior (AA), and Anal Posterior (AP).

**Figure 3.**
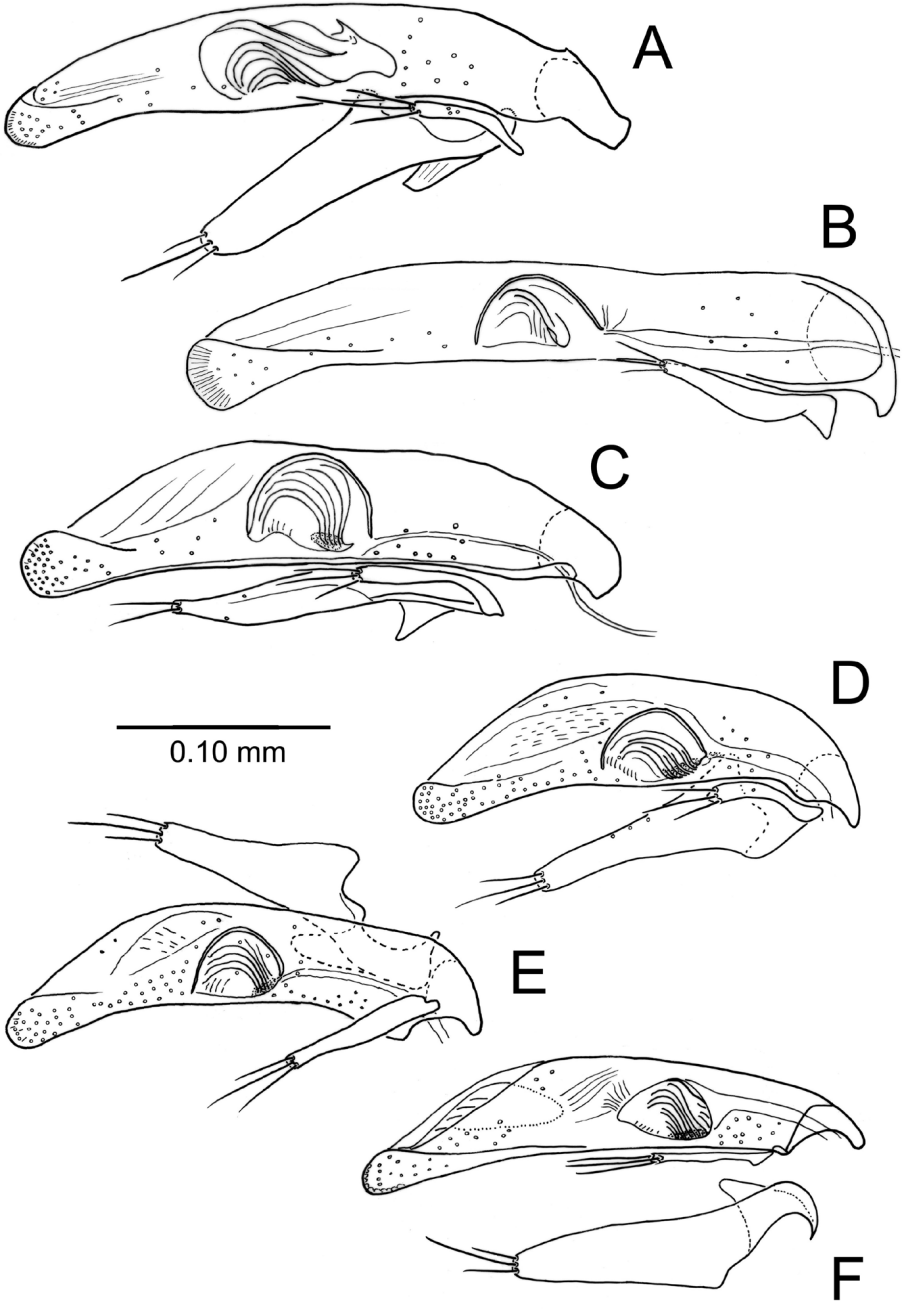
*Paratachys* spp., male aedeagus, i.e., median lobe and associated smaller right paramere and larger left paramere (right lateral view) **A***P. terryli*; Kauai: Namolokama Mtn., 23-v-2005 (CUIC) **B***P. arcanicola* lectotype slide; “*Tachys arcanicola* Blackburn Type ♂ E.C.Z. (permanent slide, Euparal?, left paramere missing; NHMUK).” **C***P. haleakalae*; East Maui: Haleakala N. P., Kipahulu Vy., 30-iv-1991 (CUIC) **D–F***P. aaa*; Hawai‘i I **D** Puna, Mountain View, Kazumura Cave, 21-xi-2018, lot HI00713 (CUIC) **E** Kau, Ocean View, Kipuka Kanohina System, 21-xi-2017, lot HI00179 (CUIC) **F** South Hilo, Kaumana Cave, 19-iii-2019, lot HI00942 (CUIC); disassociated left paramere below, ectal view.

**Figure 4.**
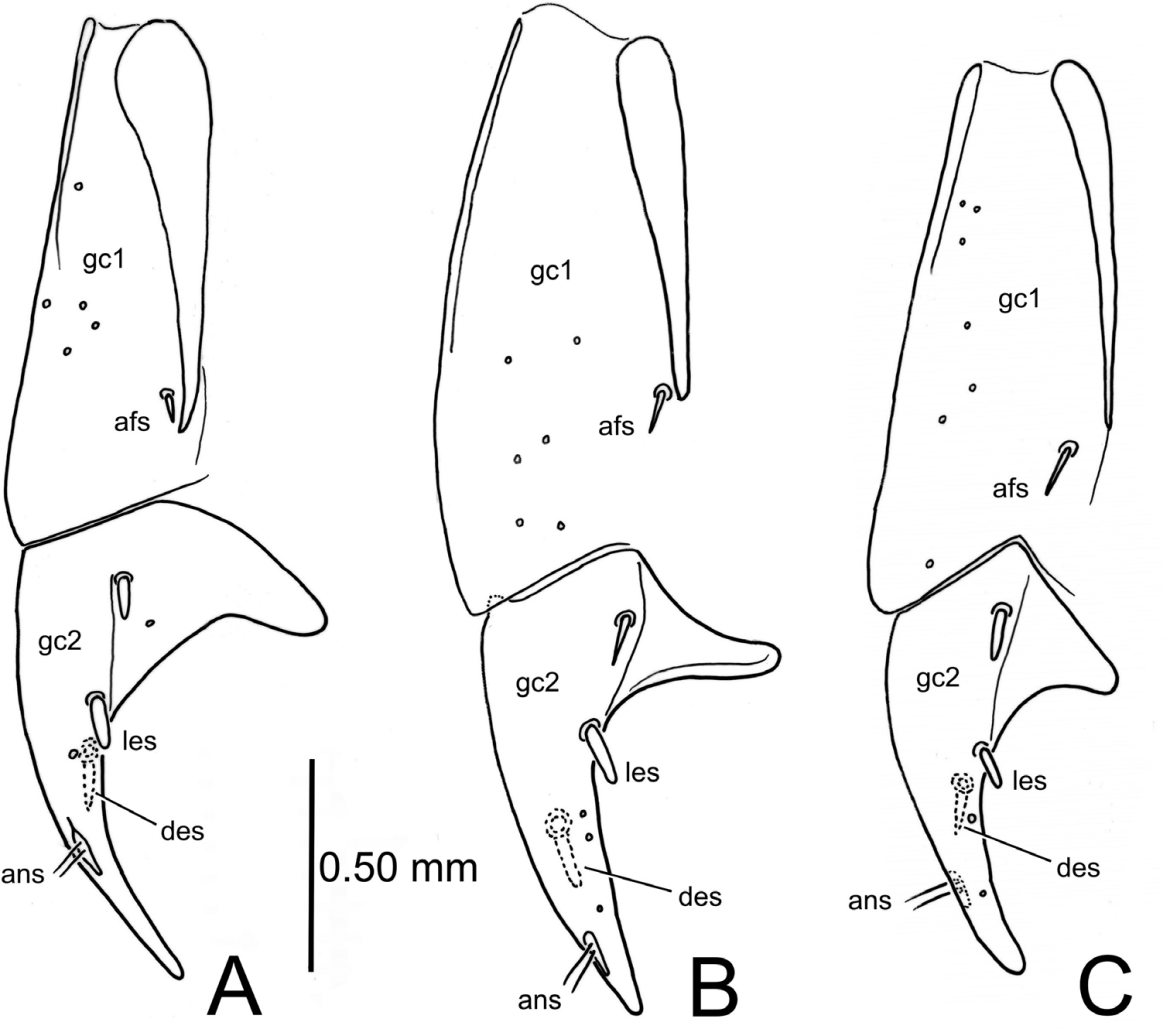
*Paratachys* spp., left female gonocoxa, ventral view **A***P. terryli*; Kauai: Namolokama Mtn., 23-v-2005 (CUIC) **B***P. haleakalae*; East Maui: Haleakala N. P., Kipahulu Vy., 30-iv-1991 (CUIC) **C***P. aaa*; Hawai‘i I.; Mountain View, Kazumura Cave, 21-xi-2018, lot HI00713 (CUIC). Abbreviations: afs, apical fringe seta; ans, apical nematiform setae; des, dorsal ensiform seta; gc1, basal gonocoxite; gc2, apical gonocoxite; les, lateral ensiform setae.

**Figure 5.**
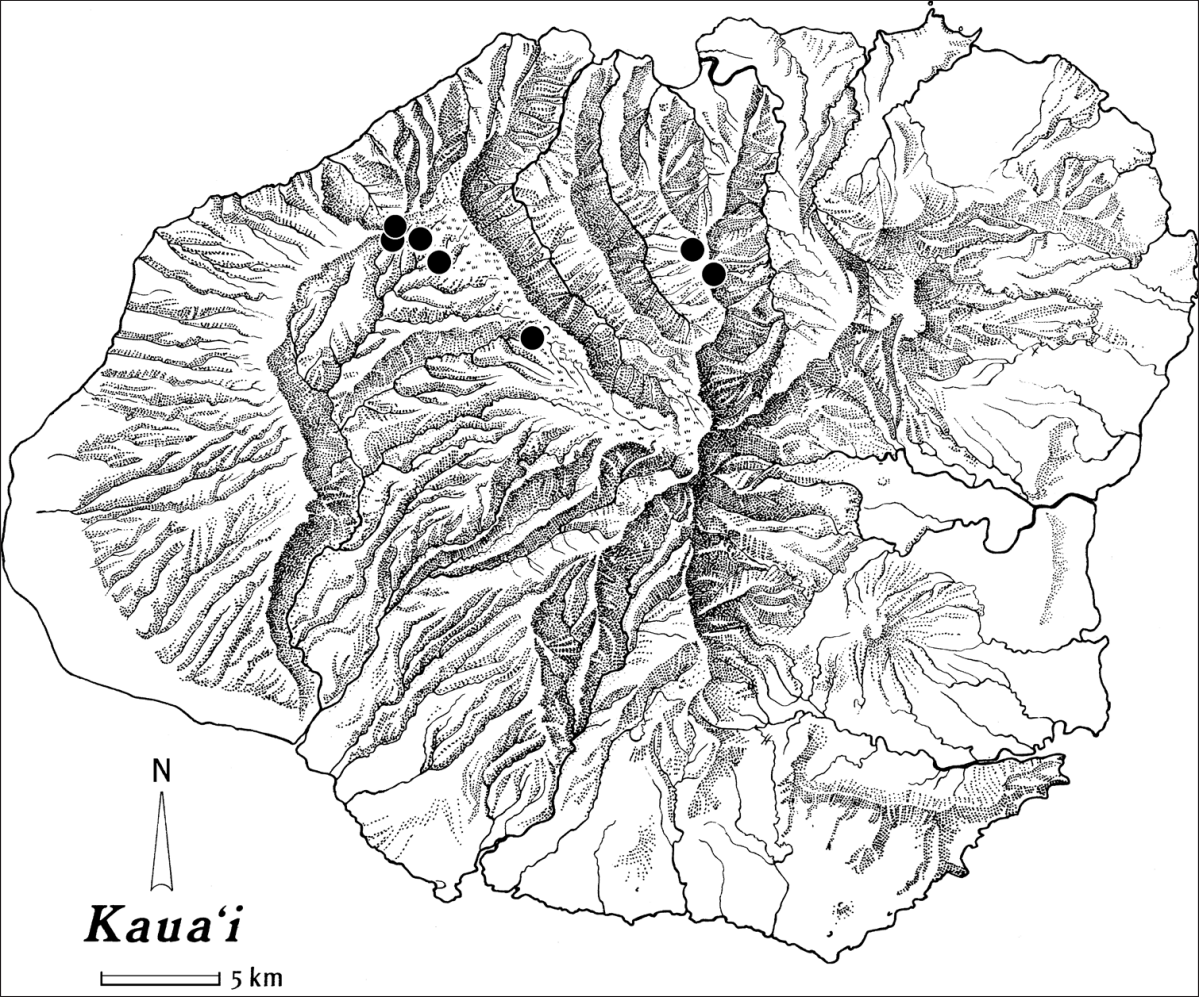
Distributional records for *Paratachys terryli*.

Figures 1 , 2A , 3A , 4A , 5

Paratachys sp. “USA: Hawaii”, Maddison et al. (2019: 162).

#### Type material.

***Holotype*** male (point-mounted, CUIC): HI: NaPali-Kona / For. Res. Kawaikoi Str. / @ Alakai Swp. Tr. 16-V- / 1991 el. 1120 m under / rocks J.K. Liebherr // HOLOTYPE ♂ / Paratachys / terryli / J. K. Liebherr 2020 (black-margined red label).

***Paratypes*. Kauai: Halelea F. R.**: Namolokama Mtn., Waioli Str., litter, sift, ohia/ferns, 22°08.00'N, 159°29.85'W, 1340 m el., 21-v-2005, lot 1 Liebherr (BPBM, 2; CUIC, 3), lot 08, Liebherr (CUIC, 1), 22°08.38'N, 159°30.18'W, 1305 m el., 23-v-2005, lot 1, Liebherr (BPBM, 2; CUIC, 3 NMNH, 2). **NaPali-Kona F. R.**: Alakai Swamp Tr. crossing Kawaikoi Str., 22°08.97'N, 159°36.95'W, 1130 m el., 14-v-1991, stop #91-24, Kavanaugh (CAS, 1), stop #91-26B (CAS, 1), Alakai Swamp Tr. E of Kawaikoi Str., leaf litter, sift/Berlese extraction, 22°08.85'N, 159°36.52'W, 1230 m el., 17-v-2005, lot 4, Liebherr (CUIC, 1), litter, sift/double boiler extraction, 22°08.85'N, 159°36.52'W, 1215 m el., 17-v-2005, lot 9, Liebherr (CUIC, 1; OSAC, 1); Mohihi Rdg. Tr., ohia forest litter, sift, moss, 22°06.83'N, 159°34.01'W, 1270 m el., 25-v-2005, lot 5, Liebherr (CUIC, 1); Pihea Trail, sifting leaf litter, 22°08.849'N, 159°37.889'W, 1218 m el., 26-vii-2015, Toledano & Olivieri (CTVR, 1), 29-vii-2015, Toledano & Olivieri (CTVR, 2), 1123 m, 29-vii-2015, Toledano & Olivieri (NHMUK, 2; CTVR, 3; CUIC, 2).

#### Diagnosis.

**Figure 6.**
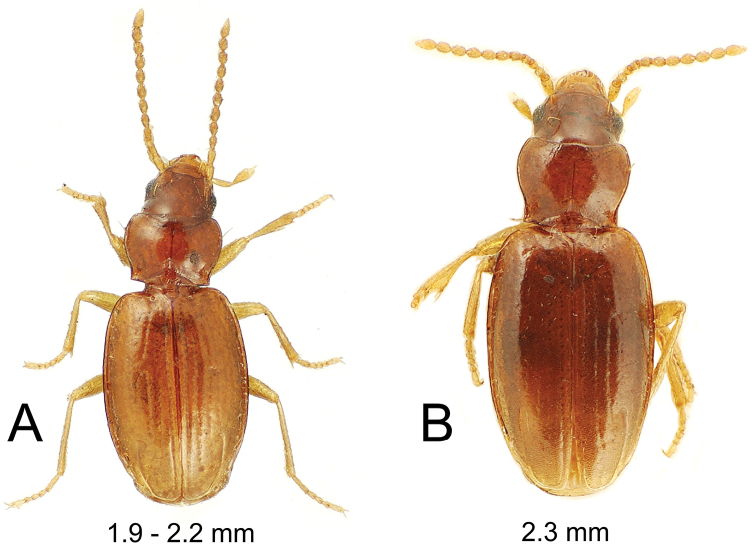
*Paratachys* spp., dorsal habitus view **A***P. arcanicola* paralectotype female; Oahu: Honolulu (Bates collection, box 389) **B***P. perkinsi* holotype female; Moloka‘i, July 1894 (R.C.L. Perkins lot 170).

This species shares elongate elytra (EL/MEW = 1.45–1.50; Fig. 1A) with *P. perkinsi* (Fig. 6B) and *P. haleakalae* (Fig. 8A), but the pronotal lateral margins are less sinuate than those of *P. perkinsi*, and more sinuate than those of *P. haleakalae*. The apical recurrent elytral groove is distinctly and narrowly impressed, with a well-defined, medially curved anterior terminus, and the elytra are truncate apically as evidenced by the nearly straight apical groove connecting the sutural and the recurrent grooves. As with *P. haleakalae*, elytral interneurs 1–3 are clearly impressed on the disc, but interneur 4 is traceable though discontinuous in this species, opposed to obsolete as in *P. haleakalae*. Standardized body length is 2.2–2.4 mm.

#### Description.

***Head*** robust, frontal grooves shallow, convergent posterad clypeus, divergent to frontal lateral margin at frontoclypeal suture just anterad antennal articulation, broadly, slightly elevated laterally to position of anterior supraorbital seta; eyes variable, from large, macrophthalmic with 12 ommatidia crossed by horizontal diameter and 15 ommatidia crossed by vertical diameter (Fig. 1C), to very small with dimensions of four ommatidia by five ommatidia horizontally and vertically (Fig. 1I), with continuous variation of dimensions between those extremes (Fig. 1D–H; see Variation section below); antennae moderately elongate, antennomere 9 ovoid, length twice diameter; labral anterior margin undulated, slightly incurved each side of midline, six-setose; penultimate maxillary palpomere broadened apically, apical palpomere a narrow spindle (Fig. 1B, E). ***Prothorax*** transverse, MPW/PL = 1.35–1.41, base moderately constricted with lateral margins sinuate anterad right hind angles, MPW/BPW = 1.21–1.34; pronotal median base depressed, longitudinally wrinkled, unmargined; basal margin beaded each side posterad laterobasal depression which is deepest just laterad broadly triangular median base; pronotal lateral margin beaded, depression mesad bead narrow but broad enough so that a row of sculpticells can be observed lining the groove; pronotal median impression finely incised, disc flat, anterior transverse impression obsolete, not evident medially or toward slightly protruded, narrowly rounded front angles. ***Elytra*** subquadrate, lateral margins evenly convex from humeri to apex, maximum width approximately midlength; basal groove present laterad position of fifth interneur, groove convexly joined to lateral marginal depression; lateral marginal depression reflexed, of equal breadth from seta Eo2 to subapical sinuation. ***Pterothorax*** elongate, mesepisternal depression smooth posterad juncture with mesosternum, depression deepest and broadest just dorsad mesocoxal cavity; metepisternum elongate, lateral length twice maximal width; metathoracic flight wings polymorphic, 18 of 19 individuals in type series with wings reduced to a vestigial stub (Fig. 1J), and 1 female with fully developed flight wings (Fig. 2A), with length of alar surface 2.3 × breadth, and radial, medial, cubital and anal veins and an oblongum cell present. ***Abdomen*** with one seta each side of apical ventrite in males, two seta each side of ventrite in females. ***Microsculpture*** evident on all somites; frons covered with evident transverse mesh, sculpticells more isodiametric posteriorly on vertex; pronotum slightly iridescent due to elongate transverse sculpticells, median base and laterobasal depressions more opaque due to reticulated isodiametric microsculpture; elytra subiridescent due to a mix of elongate transverse-mesh and transverse-line microsculpture, the transverse lines denser laterally; elytral apex more opaque due to slightly raised isodiametric sculpticells between sutural interneur and apical recurrent groove; abdominal ventrites covered with elongate transverse sculpticells, the surface glossy to subiridescent. ***Pelage*** present on head, prothorax, elytra, pterothorax, abdominal ventrites and legs; pelage on head and pronotal disc, comprising microsetae separated by distances subequal to setal length (Fig. 1F, G), as well as along ommatidial margins of the eyes (Fig. 1B); microsetae spaced slightly farther apart on elytra, with intersetal distances up to twice microsetal length; prosternum and mesosternum medially covered with microsetae as densely distributed as on frons and pronotum; pelage on abdomen present at middle of ventrite 2 between metalegs, and progressively more broadly on ventrites 3–6 (absent dorsad arc of metaleg movement); anterior surfaces of pro-, meso-, and metathoracic legs bearing pelage of elongate microsetae, the setal bases situated more closely than microsetal lengths, trochanters and coxae similarly covered with microsetae. ***Coloration*** moderately dark; vertex and frons dark brunneous to piceous, clypeus flavobrunneous, labrum flavous; basal two antennomeres flavous, outer antennomeres progressively darker, apical segments brunneous; maxillary and labial palps flavous; pronotal disc and elytra brunneous, elytral base near scutellum paler, rufobrunneous, elytral lateral marginal depression flavobrunneous, elytral epipleuron rufoflavous, contrasted to rufobrunneous thoracic ventrites; legs flavous from trochanters outward; pro- and mesocoxae concolorous with outer leg segments, metacoxae more brunneous laterally to match the dark brunneous ventrites. ***Legs*** with basal male protarsomere alone bearing a blunt, antero-apical process.

#### Variation.

The compound eyes vary dramatically in this species, from fully macrophthalmic (Fig. 1C), to microphthalmic (Fig. 1I); OR ranging 1.15–1.35 for smallest- to largest-eyed individuals. That these differences reflect infraspecific variation is supported by presence of the extremes plus a variety of intermediate configurations (Fig. 1D–H) among specimens collected microsympatrically within the identical collecting series (Fig. 1C–I). *Paratachys terryli* is also polymorphic for flight-wing development, with a single macrophthalmic female specimen (Fig. 1C) bearing fully developed flight wings (Fig. 2A). The wings of this female are of dimensions similar to those of fully flighted individuals of *Tachys oahuensis* (Fig. 2B) that were collected in ultraviolet light traps. Venation differs among the two compared species, with the wing of the female *P. terryli* exhibiting the oblongum cell but not the basal stem of the radius posterior (Kukalová-Peck and Lawrence 1993), whereas the wing of *T. oahuensis* lacks the oblongum but retains the radius posterior. Both wings are folded reflexively under the elytra, with the spring-like wing margin apicad the radial cell flipping the wing open when the wing is deployed. The various brachypterous individuals exhibit a variety of eye configurations. Wing configuration is not associated with morphological variation in elytral dimensions based on comparison of four simultaneously collected females (Namolokama Mountain, 23-v-2005, lot 1) with HuW/MEW for the macropterous female = 0.57, and HuW/MEW for the three brachypterous females = 0.57–0.58.

#### Male genitalia.

Aedeagal median lobe porrect, parallel-sided with an evenly rounded apex (Fig. 3A); flagellar complex elongate, with a sinuously scooped apical margin; right paramere narrow, strap-like with three apical setae; left paramere broad, elongate, with a broadly rounded apex and three apical setae.

#### Female reproductive tract.

Bursa copulatrix short, broad (as in Fig. 10), spermathecal duct narrow and extremely elongate, at least 0.57 mm long in single dissection attempt; gonocoxa bipartite, basal gonocoxite 1 narrow, elongate, with a broad, lateral sclerotized apodeme and a single apical fringe seta situated near apex of apodeme (Fig. 4A); apical gonocoxite falcate, broadly expanded laterally at base, apex finely acuminate; two peg-like lateral ensiform setae along mid-ventral line, and one like-sized dorsal ensiform seta dorsad the apical lateral seta; two apical nematiform setae in an elongate fossa situated at approximately 2/3 length of the apical gonocoxite.

#### Etymology.

This species honors Terry L. Erwin by combining his first name and middle initial to form the genitive patronym *Paratachys terryli*. This construction follows that of *Bembidion carlhi*Erwin and Kavanaugh (1981), a species that honors Carl H. Lindroth whose ‘Carabid Beetles of Canada and Alaska’ monograph (Lindroth 1969 et. seq.) revolutionized the study of North American Carabidae. Analogous to *B. carlhi*, this epithet recognizes Terry’s immense impact on the study of Neotropical Carabidae as well as tropical biodiversity writ large.

#### Distribution and habitat.

This species is known from the Alakai Swamp west of the Wainiha River, and from Namolokama Mountain, a ridge to the east bordered by the Lumahei and Hanalei Rivers (Fig. 5). It has been most commonly collected in leaf and moss litter taken from low stature Ohia lehua (*Metrosideros polymorpha* Gaud.; Myrtaceae) forest. At a site at 1340 m elevation on Namolokama, one sample (21-v-2005, lot 1) sifted and subsequently hand-picked contained five *Paratachys terryli* in company with two *Bembidion admirandum* (Sharp), two *B. corticarium* (Sharp), and two *Blackburnia kauaiensis* (Sharp). A second sample from the same site (21-v-2005, lot 8) was double-boiled on a stove, resulting in discovery of one *B. admirandum*, eight *B. corticarium*, and one *P. terryli*. A third Namolokama sift sample from 1305 m elevation (23-v-2005, lot 1) contained seven *P. terryli* in company with specimens of three species of *Blackburnia*; two *B. posticata* (Sharp), and one each *B. bryophila* Liebherr and *B. kauaiensis* (Sharp). Most other collections of *P. terryli* represent singletons most often collected in company with *Bembidion* spp., including: 16-V-1991 lot 5 from a rocky streambed also including five *B. ignicola* (Blackburn); 17-v-2005 lot 4 from Berlese extraction of leaf litter siftate along with two *B. admirandum* and three *B. munroi*; and 17-v-2005 lot 9, double boiled from leaf litter siftate along with six *B. admirandum* and two *Bl. posticata*. Thus, it would appear from presently available evidence that *P. terryli* occurs in microhabitats most often also occupied by *Bembidion* beetles, either along streams, or in deep terrestrial leaf litter in montane wet ohia forest.

### Paratachys arcanicola

Taxon classificationAnimaliaColeopteraCarabidae

(Blackburn)

7D3E3725-A610-584E-B16A-FC3AB9059735

Figures 3B , 6A

Tachys arcanicola Blackburn, 1878: 158; Sharp 1903: 287; Britton 1948: 240.
Paratachys arcanicola , Erwin, 1974b: 139.

#### Type material.

***Lectotype*** male (NHMUK), hereby designated: Blackburn two-parallel black line O‘ahu platen (Zimmerman 1957: 201) with glued, dissected specimen / arcani on reverse // Type (round, red-margined label) // Hawaiian Is. / Rev. T. Blackburn / 1888-30 // Tachys / arcanicola / Blackburn. Type // For / genitalia / See Type Coll. // Lectotype / Tachys / arcanicola / J. K. Liebherr 1998 (black-margined red label). The genitalia slide has left-side labels: Tachys / arcanicola / Blackburn / ♂ Type / E. C. Z. // Lectotype ♂ / Tachys arcanicola / 2020 / J. K. Liebherr (red label glued to E. C. Z. label).

***Paralectotypes***: Ins. Oahu, Ind. auth. (= Blackburn) (NHMUK, 1); O‘ahu [2-line Blackburn label], Blackburn (NHMUK, 2); Sandwich Is. (Bates collection, box 389), Blackburn (MNHN, 1). The first paralectotype listed above represents a specimen provided by the Rev. T. Blackburn to R.C.L. Perkins for his reference during the ‘Fauna Hawaiiensis’ survey.

#### Non-type material.

Honolulu, Perkins (BPBM, 2).

#### Diagnosis.

This is a small-bodied species, standardized body length 1.9–2.2 mm, with ovoid elytra; EL/MEW = 1.40. The pronotum is transverse, MPW/PL = 1.43–1.48, dimensions shared among Hawaiian *Paratachys* only with some individuals of *P. aaa* (Fig. 9A), the other small-bodied Hawaiian *Paratachys*. The pronotal lateral margins are sinuate basally, and much as in *P. terryli*, subparallel just before the nearly right hind angles. However, known specimens of this species are paler than those of *P. terryli* (Fig. 1A), with the flavobrunneous head, pronotum and elytra contrasted less with the flavous legs (Fig. 6A).

#### Description.

***Head*** quadrate, ocular lobes little projected, neck broad; frontal grooves bordering convergently convex frons that narrows from anterior supraorbital setae to clypeal margin, the grooves broad and planar laterad convex frons; clypeus convex; eyes small, only slightly convex (Fig. 6A), size ranging from five to seven ommatidia horizontally, six to nine ommatidia vertically; OR 1.16–1.26 for small-eyed vs. large-eyed individuals; labrum quadrate, apical margin straight, six-setose; antennae submoniliform, antennomere 9 length 1.6 × diameter; penultimate maxillary palpomere broadened apically, apical palpomere a narrow spindle (Fig. 6A). ***Prothorax*** transverse, MPW/PL = 1.43–1.48, base moderately constricted with lateral margins sinuate anterad right hind angles, MPW/BPW = 1.23–1.29; pronotal median base depressed, surface reticulate due to pebbly microsculpture, unmargined; basal margin beaded each side posterad laterobasal depression which is convex medially, the convexity continuous with disc; pronotal lateral margin beaded, depression mesad bead narrow, but broad enough so that a row of sculpticells can be observed lining the groove; pronotal median impression finely incised, disc convex each side of impression, anterior transverse impression shallow, defining a flat anterior collar medially, groove obsolete laterally near tightly rounded, little protruded front angles. ***Elytra*** subovoid, lateral margins evenly convex from humeri to apex, maximum width approximately midlength, HuW/MEW = 0.59; basal groove present laterad seta Ed1 (parascutellar seta), groove subangularly joined to lateral marginal depression; lateral marginal depression moderately reflexed, of equal breadth from seta Eo2 to subapical sinuation; elytral interneurs 1 and 2 deep, smooth on disc, interneur visible only as short segment anterad and obsolete very shallow impression posterad dorsal seta Ed4. ***Pterothorax*** moderately elongate, mesepisternal depression smooth posterad juncture with mesosternum, depression deepest and broadest just dorsad mesocoxal cavity; metepisternum broad and short, lateral length 1.3 × maximal width; metathoracic flight wings vestigial in six specimens examined, the broad stub extended to just beyond position of elytral seta Eo3. ***Abdomen*** with one seta each side of apical ventrite in males, two seta each side of ventrite in females. ***Microsculpture*** evident on all somites; frons covered with evident transversely stretched isodiametric mesh; pronotal disc with elongate transverse mesh, the surface slightly iridescent due to narrow elongate sculpticells, median base opaque due to rough isodiametric sculpticells; elytra subiridescent due to a mix of transverse-mesh and stretched transverse-mesh microsculpture; abdominal ventrites covered with elongate transverse sculpticells, the surface glossy. ***Pelage*** present on head, prothorax, elytra, pterothorax, abdominal ventrites and legs; pelage on head and pronotal disc, comprising microsetae separated by distances subequal to setal length, as well as along ommatidial margins of the eyes; microsetae spaced slightly farther apart on elytra, with intersetal distances up to twice microsetal length; anterior (ventral) surfaces of meso- and metathoracic legs bearing pelage of elongate microsetae, the setal bases situated more closely than microsetal lengths, trochanters and coxae similarly covered with microsetae. ***Coloration*** ferruginous (Fig. 6A); vertex and frons brunneous, clypeus flavo-brunneous, labrum flavous; antennae flavous; maxillary and labial palps flavous; pronotal disc and elytra flavo-brunneous, elytral lateral marginal depression flavous, elytral epipleuron flavo-brunneous, contrasted to rufo-flavous thoracic and abdominal ventrites; legs flavous from trochanters outward; pro- and mesocoxae concolorous with outer leg segments, metacoxae rufo-flavous to match abdominal ventrites.

#### Male genitalia.

Aedeagal median lobe nearly straight for much of length, slightly, evenly downturned to the broad, shovel-nosed tip (Fig. 3B); flagellar complex hemi-ovoid, the scoop-like apical surface only slightly incurved along ventral margin; strap-like right paramere with three apical setae [left paramere lost from dissection].

#### Distribution and habitat.

The Reverend Thomas Blackburn (1878) noted in his initial description of this species, “Very local, but not rare in some mountainous localities (Blackburn 1878: 158).” Nonetheless, Britton (1948: 240) noted only three specimens in the Blackburn collection (NHMUK), listing them as found at “1500 ft., under bark.” The label data do not confirm the elevation or situation, though Blackburn did write “I spent some portion of time (varying from an hour to an occasional twelve hours) in collecting insects on the eastern side of Oahu, as nearly as possible once a fortnight on the average through the six years I spent on the Hawaiian Islands (Blackburn 1885: 204).” Such relatively brief forays would have restricted his collecting to the southern Koolau Range, encompassing the hills above Honolulu including Round Top, Tantalus, or perhaps Lanihuli or Konahuanui on a long day. These areas were considerably more accessible at that time due to the absence of human residential developments and the depredations of goats on the native vegetation that opened the forest (Liebherr and Polhemus 1997). R.C.L. Perkins collected two specimens at Honolulu in the early 20^th^ Century, also very likely found on or near Tantalus, as he frequented that site in a continuing search for rarely collected insects (Perkins 1906), correctly predicting the extinction of many species found only there (e.g., Liebherr 2009). No specimens have been collected near “Honolulu” since Perkins, and we can surmise that this species is extinct.

#### Nomenclatural notes.

Erwin (1974b) clarified generic concepts of New World tachyines, assigning species to proper genera, and designating lectotypes to stabilize nomenclature for type series of species deposited among multiple worldwide institutions. He assigned *Tachys arcanicola* Blackburn to *Paratachys* Casey and wrote “Lectotype, male, here designated, in NHMUK (Erwin 1974b: 139).” Previously, Britton (1948: 240) cited three NHMUK Blackburn specimens from Oahu, all males, as representatives of *T. arcanicola*, although he did not select a lectotype from among them. Later during his 1948–1972 tenure as an honorary associate of The Natural History Museum, London (Upton 2004), E.C. Zimmerman dissected the male labelled with the round, red-margined “Type” label during his researches on Hawaiian Carabidae, with the dissection described above. The NHMUK round red-margined “Type” labels were used variously to designate specimens prior to World War II, and then all beetle specimens were dispersed to safe haven in Wales, Scotland, and to the west of London prior to the Nazi Blitz of 1940–1941 (Jones 2013; M.E. Bacchus, pers. comm.). Thus, the labels themselves do not hold nomenclatural value. In 1998, the author examined the three male specimens reported in Britton (1948), and labelled the specimen dissected by Zimmerman and bearing the red-margined “Type” label as lectotype. No other specimen of *T. arcanicola* bearing an Erwin lectotype label was found among NHMUK material at that time. This absence was affirmed by a recent search of the entire NHMUK tachyine holdings (B. Garner, pers. comm.), again resulting in no Erwin-labelled lectotype specimen for this taxon, though Erwin labels were found on various other specimens representing Neotropical taxa for which lectotypes were designated in Erwin (1974b). More broadly, no Erwin lectotype-labelled specimen was found by the author in the H. W. Bates collection in the MNHN, Paris, although a single specimen in the Bates holdings of the Oberthür Collection is accounted for above as a Parisian paralectotype. Because all three syntype specimens of this species in the British Museum are males, all three were identically accounted for both in Britton (1948) and during the author’s 1998 visit, and no other Erwin-labelled lectotype specimen exists in Bates material in Paris, it is unavoidably concluded that Erwin never labelled a specimen as lectotype for this taxon. Because his published designation specified a male specimen, but there are three male syntype specimens in the NHMUK, Erwin failed to designate a “single name-bearing type specimen” as lectotype (I.C.Z.N. 1999, glossary). As “A lectotype may be designated from syntypes to become the unique bearer of the name of a nominal species-group taxon (I.C.Z.N. 1999, Article 74.1)”, his published statement is invalid. In the extremely unlikely circumstance that an Erwin-labelled lectotype specimen would surface from some other venue, unlikely because it cannot be one of the three NHMUK specimens cited by Britton (1948), and its very existence would violate Erwin’s (1974b) statement that his lectotype was designated from a NHMUK male specimen, then the lectotype presently designated above would become invalid. The present lectotype designation is undertaken to eliminate ambiguity with regard to how this species is to be tied to zoological nomenclature, with future rediscovery of an Erwin-labelled lectotype specimen of a Blackburn syntype the only means to validate Erwin’s (1974b) published action.

### Paratachys perkinsi sp. nov.

Taxon classificationAnimaliaColeopteraCarabidae

6BF82F1F-8040-555E-A395-709A4683182F

http://zoobank.org/625B85AF-A675-44A2-BA51-0B6B502695D4

Figures 6B , 7

Tachys arcanicola , Britton 1948: 240 (misidentification).

#### Type material.

***Holotype*** female (NHMUK): platen mount / 170 on reverse // Hawaiian Is. / R.C.L. Perkins // Kaunakakai / sea level / vii-93 // atomarium (label upside down indicating misidentification) // HOLOTYPE ♀ / Paratachys / perkinsi / J. K. Liebherr 2020 (black-margined red label). Britton (1948: 240) lists a second specimen with these data; however, only the single specimen designated holotype above was observed by the author in 1998 (unpubl. data).

#### Diagnosis.

Distinguished among all Hawaiian *Paratachys* by the elongate sinuation of the pronotal lateral margins before the right hind angles; the subquadrate, elongate elytra; and the angulate humeral juncture of the basal and marginal elytral grooves (Fig. 6B). The single known specimen has convex eyes with ten ommatidia crossed by a horizontal diameter of the eye, and 12 ommatidia crossed by a vertical diameter; the OR is 1.26. Elytral interneurs 1 and 2 are continuous on the disc, and interneur 3 is deepest in the basal 1/4 of elytral length and discontinuous on the disc. Standardized body length 2.3 mm.

#### Description.

***Head*** appearing narrow due to elongate mandibles, mandibular length from dorsal condyle to apex twice distance from condyle to lateroapical angle of labrum; ocular lobes little projected, neck broad, eyes convex but small (Fig. 6B); frontal grooves bordering convergently convex frons that narrows from anterior supraorbital setae to clypeal margin, the grooves broad and planar laterad convex frons; clypeus convex; labrum transverse, broadly, slightly emarginate apically, six-setose; antennae submoniliform, antennomere 9 length 1.6 × diameter; penultimate maxillary palpomere broadened apically, apical palpomere a narrow spindle (Fig. 6B). ***Prothorax*** slightly transverse, MPW/PL = 1.41, base moderately constricted with lateral margins sinuate well before acute hind angles, MPW/BPW = 1.21; pronotal median base depressed relative to disc, the juncture of disc and base smooth, surface of median base slightly roughened due to minute lenticular longitudinal wrinkles; basal margin trisinuate, medially incurved, laterally slightly oblique, a thin marginal bead posterad laterobasal depressions; depressions deepest along a line running from hind angles parallel to median base-discal margin (Fig. 6B); pronotal lateral margin beaded, the bead directly adjacent to pronotal disc at midlength; pronotal median impression finely incised, disc convex each side of impression; anterior transverse impression shallow medially, well defined laterally as a narrow groove that extends to moderately projected, tightly rounded front angle. ***Elytra*** elongate, broad basally, EL/MEW = 1.57, HuW/MEW = 0.64, lateral margins evenly convex from humeri to subapical sinuation; basal groove evident from fourth interneur, juncture with lateral marginal depression subangulate; lateral marginal depression moderately reflexed, surface translucent, of equal breadth from seta Eo3 to subapical sinuation; disc flat between third interneurs each side; interneurs 1 and 2 deep on disc, slight irregularities along deepest portions, interneur 3 evident anterad and posterad dorsal seta Ed4, the setal impression obscuring interneur near seta. ***Pterothorax*** elongate, mesepisternal depression smooth posterad juncture with mesosternum, depression deepest and broadest just dorsad mesocoxal cavity; metepisternum lateral length 2 × maximal width; metathoracic flight wings broad vestigial flaps that extend to position of elytral seta Eo3 (visible in holotype through translucent elytra). ***Abdomen*** with two setae each side of apical ventrite in female holotype. ***Microsculpture*** evident on all somites; frons covered with evident transversely stretched isodiametric mesh, sculpticells more isodiametric in frontal grooves; pronotal disc with elongate transverse mesh, the surface slightly iridescent due to narrow elongate sculpticells, median base opaque in depressions, surface glossy along elevated ridges; elytra subiridescent due to a mix of transverse-mesh and stretched transverse-mesh microsculpture. ***Pelage*** on head, pronotal disc and elytra comprising microsetae separated by distances twice setal length; short microsetae along ommatidial margins of eyes; anterior (ventral) surface of meso- and metathoracic legs bearing pelage of elongate microsetae, the setal bases situated more closely than microsetal lengths. ***Coloration*** ferruginous (Fig. 6B); vertex and frons brunneous, clypeus flavo-brunneous, labrum, mandibles and palpomeres flavous; antennae flavous; pronotal disc and elytra flavo-brunneous, elytral lateral marginal depression slightly paler, elytral epipleuron flavo-brunneous, contrasted to rufo-flavous thoracic and abdominal ventrites; legs flavous from trochanters outward; pro- and mesocoxae concolorous with outer leg segments.

#### Female reproductive tract.

The single historically collected holotype specimen was not dissected.

#### Etymology.

This species is named to honor its collector, R.C.L. Perkins, who as a new graduate of Oxford University was sent in 1891 to Hawai‘i, by the British Association for the Advancement of Science to collect zoological specimens in support of the ‘Fauna Hawaiiensis’ project (Sharp 1913; Manning 1986). Dr. Perkins stayed on after his initial Hawaiian surveys, during which the holotype of this species was collected, to serve in the Territory of Hawaii’s Agricultural Department and then as Director of the Experiment Station of the Hawaiian Sugar Planters’ Association (Scott and Benson 1955; Scott 1956). Throughout his scientific career, Perkins conducted systematic research on a wide variety of insect taxa (Evenhuis 2005) as well as the control of pestiferous insects and weeds using introduced natural enemies (Perkins 1897, 1923; Perkins and Swezey 1924).

#### Distribution and habitat.

**Figure 7.**
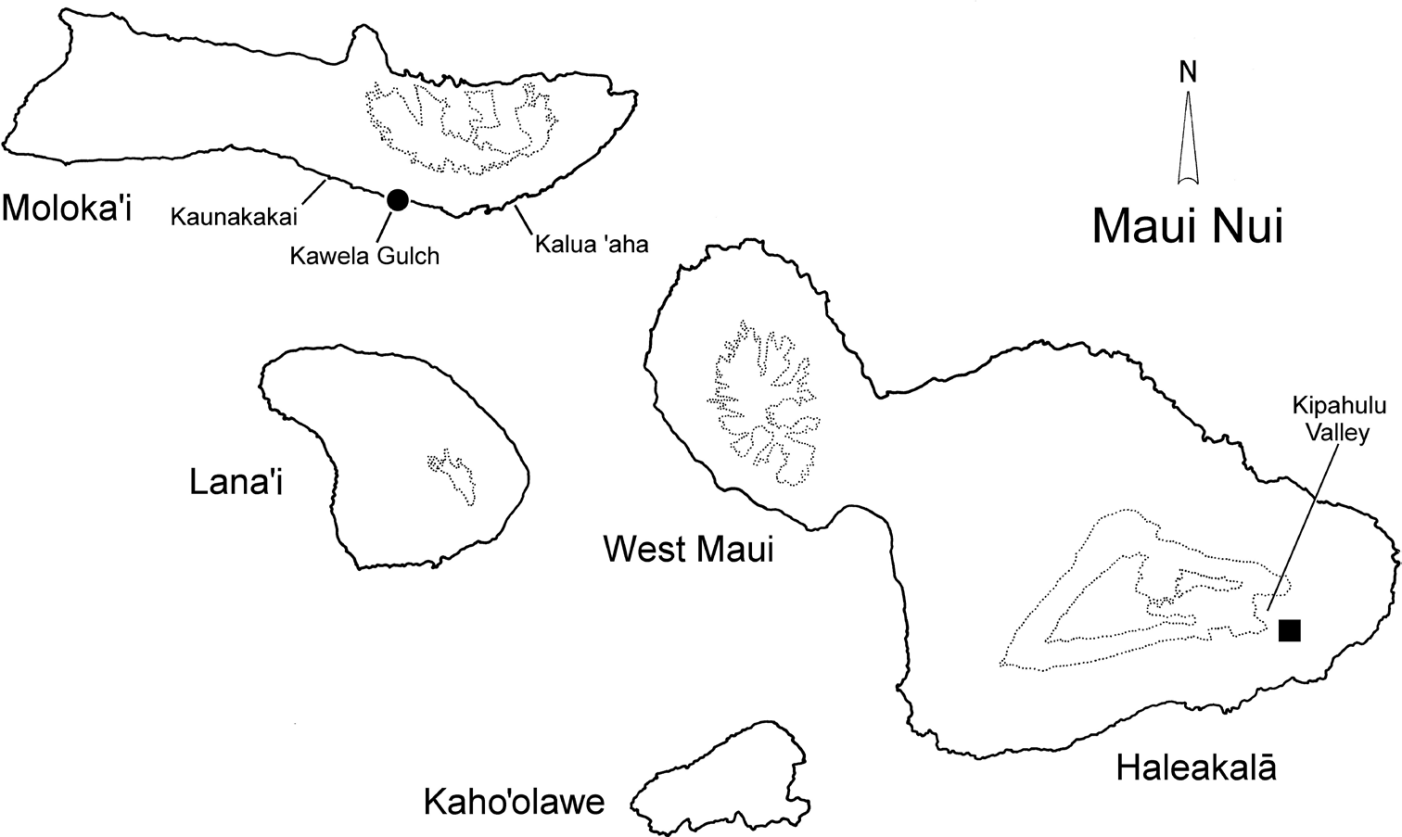
Distributional records for *Paratachys* spp.; *P. perkinsi* (type locality designated as Kawela Gulch at sea level (•) (see text); *P. haleakalae* (▪).

The lone specimen representing this species is labelled as Perkins’ lot 170, Kaunakakai, July 1893, sea level. Perkins’ Moloka‘i July collecting commenced on 9 July, however much of his time was spent high in the forests (Evenhuis 2007). He walked to the coast on 17 July, arriving at Kaunakakai in the afternoon. He spent the next two days hunting shorebirds and coots, and on the 20^th^ “Walked along the coast to Kaluaaha supposed to be about 16 miles E. ... I stayed some time at Kawela ... (Evenhuis 2007: 262).” After his midday stop at the base of Kawela Gulch he encountered a “heavy shower” and “finishing of the school term” at Kalua ‘aha. The next day he walked back to Kaunakakai, and on the 21^st^ came down with a sore throat, which progressed to what he self-diagnosed as “the ‘grippe’ or influenza (Evenhuis 2007: 163).” He returned to the mountains on 24 July, closing the temporal window during which *P. perkinsi* could have been collected. Thus, a best guess concerning the collecting locality of the lone *Paratachys perkinsi* is in the streambed of Kawela Gulch near the coast on 20 July (Fig. 7). Such a situation would be similar to the streamside situations frequented by *P. terryli* of Kauai, although at considerably lower elevation. Kawela Stream at higher elevations houses several flightless riparian species, including the nabid bug, *Nabis gagneorum*Polhemus (1999), and the very large-bodied, vestigially winged carabid beetle, *Blackburnia polhemusi* Liebherr (Liebherr and Zimmerman 2000). The riparian habits of both of these species are unusual among their respective radiations, pointing to Kawela Gulch as a persistent and stable water source able to support populations of flightless insects.

### Paratachys haleakalae sp. nov.

Taxon classificationAnimaliaColeopteraCarabidae

184DDEAA-5719-5D15-B734-0C938CA489AA

http://zoobank.org/59217277-0305-4456-89D2-27B4DDE94649

**Figure 8.**
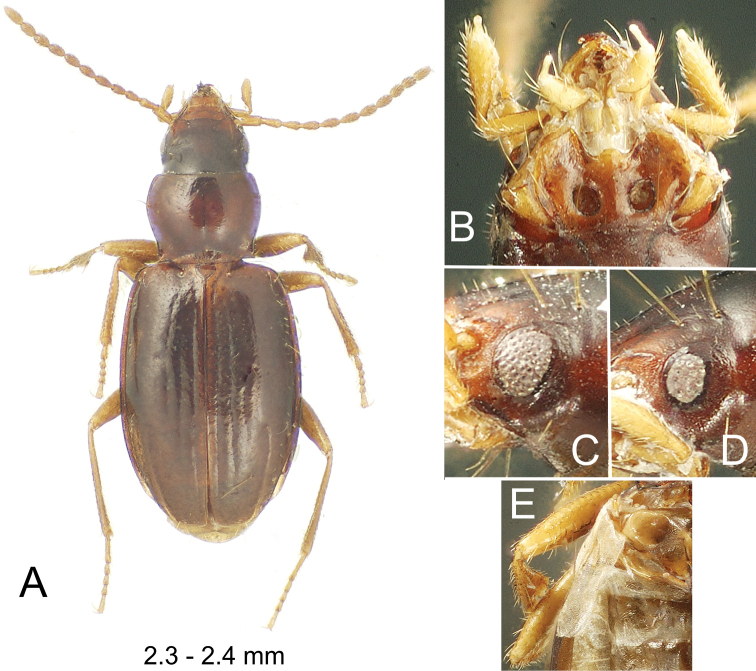
*Paratachys haleakalae***A** dorsal habitus view **B** mentum and associated mouthparts, ventral view **C** larger compound eye of female, lateral view **D** smaller compound eye of male; lateral view **E** stenopterous left metathoracic flight wing of female (elytron removed), dorsal view.

Figures 3C , 4B , 7 , 8

#### Type material.

***Holotype*** female (CUIC; undissected, point-mounted): HI:Maui Haleakala N.P. / Kipahulu Vy. Central / Pali Tr. 910 m el. / 30-IV-1991 under logs // J.K. Liebherr / A.C. Medeiros / Jr. collectors // HOLOTYPE ♀ / Paratachys / haleakalae / J. K. Liebherr 2020.

***Paratypes*.** Three specimens with same collection data as holotype (CUIC, 3; dissected male allotypic paratype and two female paratypes, one lacking left elytron; Fig. 8E).

#### Diagnosis.

Immediately diagnosable among Hawaiian *Paratachys* by the narrow, more quadrate pronotum that is little constricted basally (Fig. 8A); MPW/PL = 1.29–1.36 and MPW/BPW = 1.21–1.26. The pronotal basal margin is shallowly sinuate just inside the hind angles, with the median basal margin extended posteriorly as a narrow collar. Elytral interneurs 1 and 2 are deep and broad on the disc, and interneur 3 is evident posterad dorsal seta Ed4. The eyes are somewhat variable, but always small and little convex; 4–7 ommatidia crossed by a horizontal diameter of the eye, and 6–9 ommatidia crossed by a vertical diameter (Fig. 8C, D). The head, pronotum and elytra are a rich rufobrunneous, distinctly contrasted to the flavous antennae and legs. Standardized body length 2.3–2.4 mm.

#### Description.

***Head*** narrow, ocular lobes little projected (Fig. 8A), eyes small and little convex, OR = 1.14 for individuals with small eyes (Fig. 8D) vs. OR = 1.20 for those with large eyes (Fig. 8C); frontal grooves broadly depressed, slightly convergent from position of anterior supraorbital seta to frontoclypeal suture, not extended along suture, frons only slightly convex; antennae moderately elongate, antennomere 9 length twice diameter; mandibles elongate, distance from dorsal condyle to apex twice distance from condyle to anterolateral labral margin; labrum quadrate, anterior margin evenly emarginate across width, six-setose; penultimate maxillary palpomere broadened apically, apical palpomere a narrow spindle (Fig. 8A, B). ***Prothorax*** narrow, quadrate, lateral margins subparallel anterad right to slightly acute hind angles; depressed pronotal median base narrow, juncture with disc smooth, surface with irregular longitudinal wrinkles, basal margin smooth medially, only a very narrow marginal bead laterally along sinuosity behind laterobasal depressions; laterobasal depression deepest, pit-like just laterad median base; pronotal median impression finely incised to obsolete on flattened disc; anterior transverse impression obsolete medially and laterally toward narrowly rounded, not projected front angles. ***Elytra*** subquadrate, narrow, humeri rounded, EL/MEW = 1.53; basal groove present laterad position of fourth interneur, groove convexly joined to lateral marginal depression; lateral marginal depression moderately reflexed, of equal breadth from seta Eo2 to subapical sinuation. *Pterothorax* elongate, mesepisternal depression smooth posterad juncture with mesosternum; metepisternum elongate, lateral length 1.9 × maximal width; metathoracic flight wings vestigial, the alae reduced to stenopterous flaps that extend to posterior margin of first abdominal tergite (Fig. 8E), vestiges of radial and medial veins visible in the wing membrane. ***Abdomen*** with one seta each side of apical ventrite in male, two seta each side of ventrite in females. ***Microsculpture*** evident on all somites; frons covered with evident transverse mesh, sculpticells more isodiametric in broad frontal grooves; pronotum slightly iridescent due to elongate transverse sculpticells, median base with isodiametric sculpticells in wrinkles, elevated ridges glossy; elytra subiridescent due to a mix of elongate transverse-mesh and transverse-line microsculpture, the transverse lines denser laterally; abdominal ventrites iridescent, covered with swirling, elongate transverse sculpticells. ***Pelage*** present on head, prothorax, elytra, pterothorax, abdominal ventrites and legs; pelage on head and pronotal disc comprising microsetae separated by distances subequal to setal length (Fig. 8C, D), as well as along ommatidial margins of the eyes (Fig. 8B); microsetae spaced slightly farther apart on elytra, with intersetal distances up to twice microsetal length; prosternum and mesosternum covered with sparse pelage medially; pelage on abdomen present at middle of ventrite 2 between metalegs, and progressively more broadly on ventrites 3–6 (absent dorsad arc of metaleg movement); anterior surface of prothoracic femora sparsely covered with microsetae, anterior (ventral) surfaces of meso- and metathoracic legs more densely covered with elongate microsetae, the setal bases situated more closely than microsetal length, trochanters and coxae similarly covered with microsetae. ***Coloration*** moderately darkened; vertex and frons dark brunneous to piceous, clypeus flavo-brunneous, labrum flavous; antennomeres flavous, outer antennomeres 4–11 with slight smoky cast; maxillary and labial palps flavous; pronotal disc rufo-brunneous, median base paler, rufo-flavous; elytra dark brunneous, paler near scutellum and on humeri, sutural interval may be slightly paler, elytral lateral marginal depression rufo-flavous; elytral epipleuron rufo-flavous, concolorous with thoracic ventrites; abdominal ventrites dark brunneous medially, paler laterally; legs flavous from trochanters outward; pro- and mesocoxae concolorous with outer leg segments, metacoxae brunneous to match thoracic ventrites. ***Legs*** with basal male protarsomere alone bearing a blunt, antero-apical process.

#### Male genitalia.

Aedeagal median lobe convex dorsally, ventral margin straighter, apex a broadly rounded knob densely covered with sensilla (Fig. 3C); flagellar complex broadly hemi-ovoid, apically covered with radiating cuticular ridges; right paramere narrow with three apical setae; left paramere moderately broadened, apically narrowed, the narrow apex with two setae.

#### Female reproductive tract.

Bursa copulatrix short, broad, with spermathecal duct basal terminus at juncture of bursa and common oviduct (as in Fig. 10); spermathecal duct elongate, at least 0.64 mm (duct broken in single attempted dissection); gonocoxa bipartite; basal gonocoxite 1 elongate, with a single apical fringe seta near apex of lateral apodeme (Fig. 4B); apical gonocoxite 2 broadened basally, with lateral extension directed dorsad to ventral surface of coxite bearing the two lateral ensiform setae; one dorsal ensiform seta situated halfway between position of apical lateral seta and narrow, pointed apex; two apical nematiform setae situated in elongate fossa near apex of gonocoxite.

#### Etymology.

The species epithet *haleakalae* represents the first declension genitive form indicating the species is distributed on Haleakalā Volcano, East Maui.

#### Distribution and habitat.

The type locality of *P. haleakalae* is Dogleg Camp, so named because the site is near a prominence of the upper shelf of Kipahulu Valley at 3000 ft. (910 m) elevation, 20°42.03'N, 156°04.93'W, that overlooks the lower part of the valley drained by Palikea Stream (Fig. 7). The forest at Dogleg Camp is montane wet forest dominated by large *Acacia koa* A. Gray (Fabaceae), with lower stature *Cheirodendron trigynum* (Gaud.) A. Heller, and woody herbaceous *Broussasia arguta* Gaud. (Hydrangeaceae), *Clermontia* Gaud. (Campanulaceae), *Hedyotis* L. (Rubiaceae), *Pelea* A. Gray (Rutaceae), and *Scaevola* L. (Goodeniaceae) also present. The climbing vine-like *Freycinetia arborea* Gaud. (Pandanaceae) is also quite common. That said, the type series of *P. haleakalae* was collected from under a koa log round serving as a step on the pathway to the camp privy. The beetles were in voids amongst plant roots under the log round. This is perhaps the author’s sole datum discounting the Darlington Rule: “Never roll rocks in the tropics (P.J. Darlington, Jr., pers. comm.).”

### Paratachys aaa sp. nov.

Taxon classificationAnimaliaColeopteraCarabidae

0C00EF7E-1F3C-5F19-B3D0-9B6A6653C622

http://zoobank.org/9985664D-8441-4E08-BE2F-AA1AE24BE8C2

**Figure 9.**
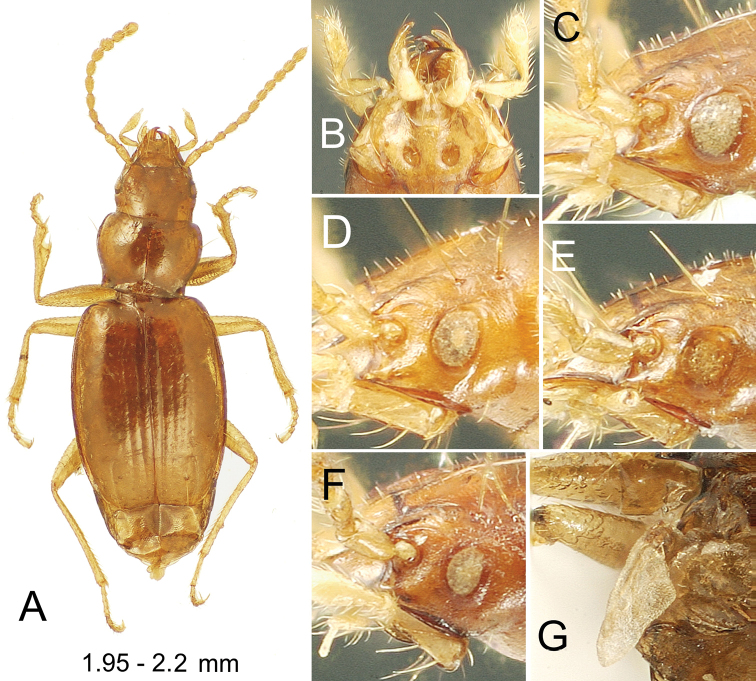
*Paratachys aaa***A** male, dorsal habitus view; Kau, Kona Mala, 21–xi–2017, lot HI00179 **B** mentum and associated mouthparts, ventral view, of above male specimen **C** larger ovoid compound eye of female, lateral view; Mountain View, Kazumura Cave, 21–xi–2018, lot HI00722 **D** moderately sized ovoid compound eye of male, lateral view; also lot HI00722 **E** smaller rounded compound eye of male, lateral view; Kaimu, Burn Cave, 16–19-iii-1994 **F** small compound eye of male, lateral view; Kaimu, Burn Cave, 16-iii-1994 **G** stenopterous left metathoracic flight wing of male (elytron removed), dorsal view; Kau, Kona Mala, 21-xi-2017, lot HI00179.

**Figure 10.**
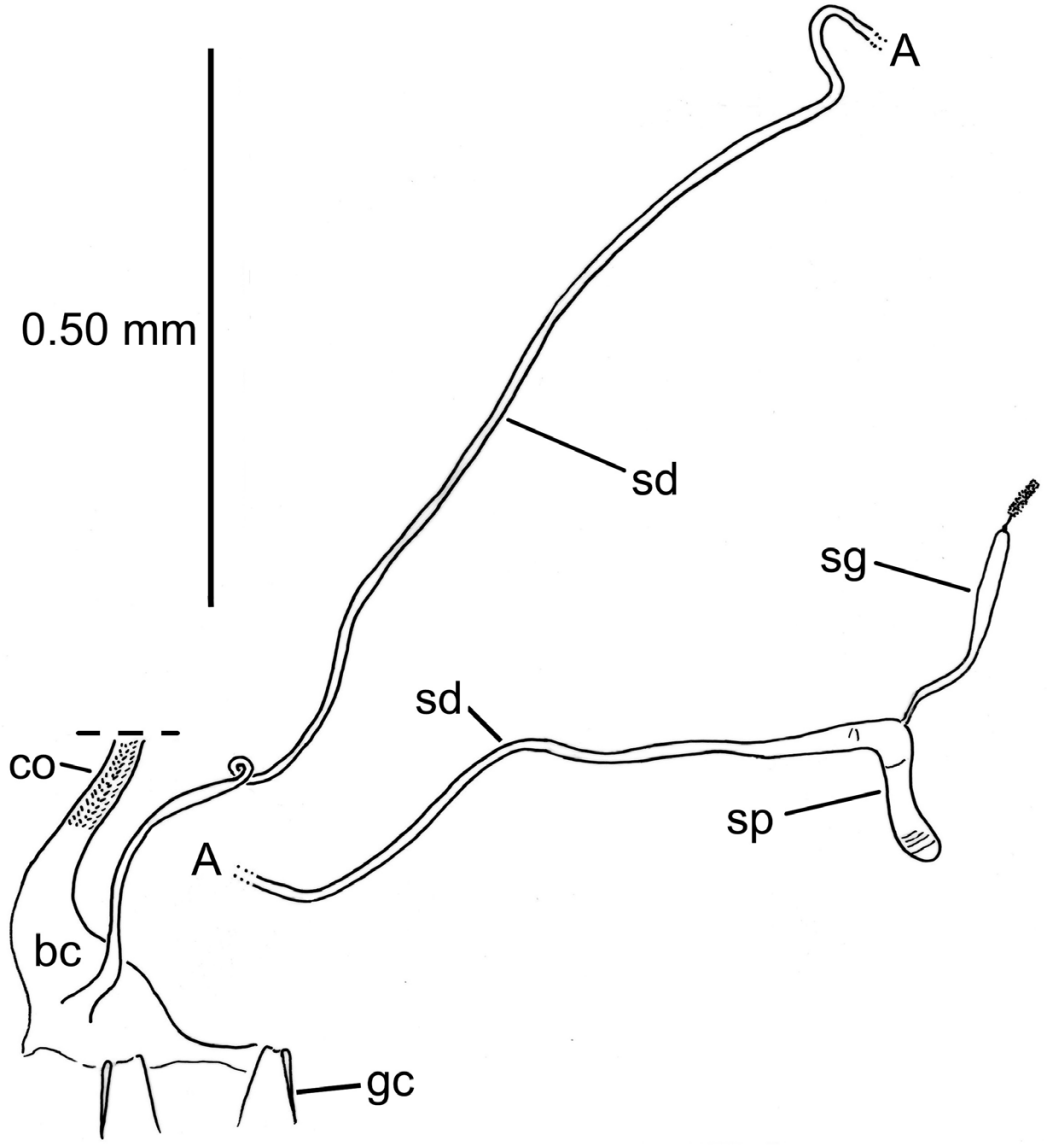
*Paratachys aaa* female reproductive tract, ventral view; Mountain View, Kazumura Cave, 21-xi-2018, lot HI00713 (CUIC). Abbreviations: bc, bursa copulatrix; co, common oviduct; sd, spermathecal duct; sg, spermathecal gland; sp, spermatheca. Schematic drawing breaks spermathecal duct at “A” to accommodate drawing to page size. Estimated length of spermathecal duct 1.7 mm.

**Figure 11.**
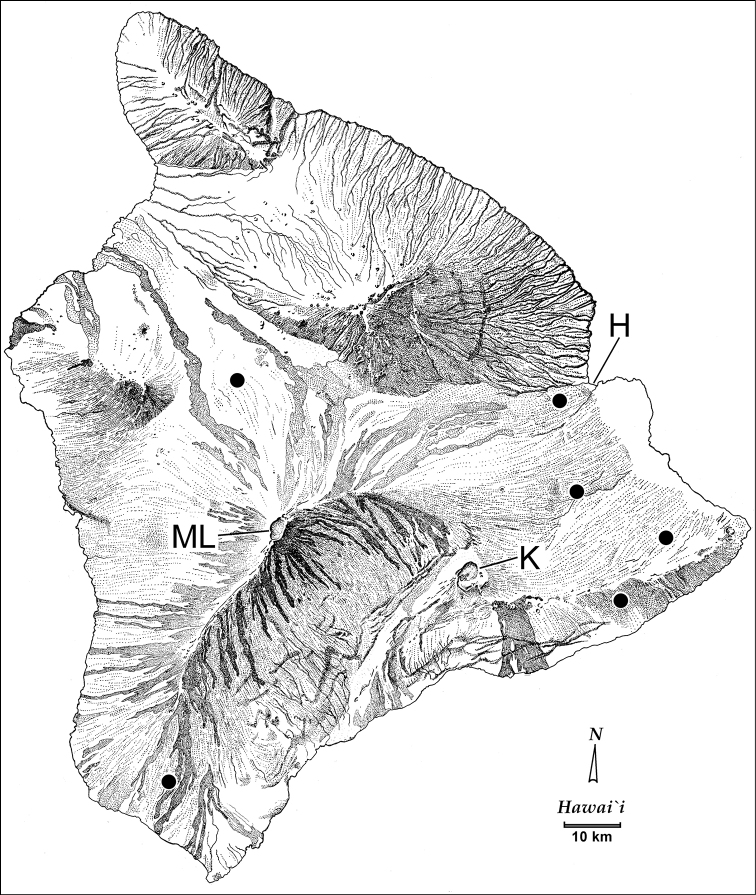
Distributional records of *Paratachys aaa*. Abbreviations: H, Hilo; K, caldera of Kilauea Volcano; ML, summit caldera of Mauna Loa Volcano.

Figures 3D–F , 4C , 9 , 10 , 11

#### Type material.

***Holotype*** male (BPBM): HI: Hawaii Mountain View / Kazumura Cave 22-VII- / 1971 F. G. Howarth / 600’ Inside lava tube cave / on slime on wall // BBM-00301 // HOLOTYPE ♂ / Paratachys / aaa / J. K. Liebherr 2020 (black-margined red label).

***Allotypic paratype*** female (BPBM): HI: Hawaii Mountain View / Kazumura Cave 25-VII- / 1971 F. G. Howarth / 200’ Inside lava tube cave // BBM-00302 // ALLOTYPE ♀ / Paratachys / aaa / J. K. Liebherr 2020 (black-margined red label).

***Paratypes*. Hawai‘i I.: Hamakua District**: Pohakuloa Military Training Area, Bobcat Trail, Cave 10265DE, deep zone, 1650 m el., 30-xii-1994, Howarth (BPBM, 3). **Kau District**: Ocean View, Kipuka Kanohina System, Cordwinder Natural Bridge, upslope, 23-xi-2018, Hackell/ Porter/ Hudson/ Katz, lot HI00776 (UHIM, 1), Kona Mala Driveway Entr., 22-xi-2016, M. Slay/ C. Slay/ Porter, lot HI00094 (BPBM, 1; UHIM, 2), 21-xi-2017, M. Slay/ C. Slay/ Gracanin/ Hackell/ A. Engel/ S. Engel/ Porter, lot HI00179 (CUIC, 1), 25-xi-2017, C. Slay/ A. Engel/ Hackell, lot HI00301 (UHIM, 3), Kula Kai Caverns, Chocolate Factory, 22-xi-2017, Porter/ S. Engel/ A. Engel/ Bosted, lot HI00227 (UHIM, 1), Menehune Entrance, 11-xi-2018, Chong/ Hudson/ Porter/ Thomson, lot HI00508 (UHIM, 1), 20-xi-2018, M. Slay/ S. Engel/ Katz/ Taylor (UHIM, 3), Wilson’s Big Room, 20-xi-2018, C. Slay/ Engel/ Katz/ Taylor, HI00673, lot HI00619 (UHIM, 2), Xanadu XD 1, 23-xi-2016, M. Slay/ C. Slay/ Porter, lot HI00142 (UHIM, 1), Xanadu Extension 1, 23-xi-2018, M. Slay/ C. Slay/ Yelverton/ Gunter/ Gracanin, lot HI00755 (UHIM, 1). **Puna District**: Kaimu, Burn Cave, deep zone, site 4, 260 m el., 16-iii-1994, Howarth/ Miller (BPBM, 1), pitfall trap L-8, 260 m el., 16–19-iii-1994, Howarth/ Miller (BPBM, 1), pitfall trap M-5, 16–19-iii-1994, Howarth/ Miller (BPBM, 2); Mountain View, Cow’s Eye Entrance, 22-iii-2019, Hudson/ Gracanin/ Hackell/ C. Slay/ M. Slay, lot HI01050 (UHIM, 2), lot HI01061 (BPBM, 3), D Road Cave, 20-iii-2019, Porter/ Chong/ Engel/ Hudson/ Hackell, lot HI00973 (UHIM, 2), lot HI00988 (BPBM, 2), Epperson’s Cave, 20-iii-2019, C. Slay, M. Slay, Engel, Gracanin, lot HI 00949 (UHIM, 1), Keala Cave Entrance, 19-iii-2019, A. Engel, S. Engel, Hudson, Hackell, M. Slay, C. Slay, Porter, Gracanin, lot HI 00911 (UHIM, 1), Kazumura Cave, lava tube cave, 200’ inside lava tube cave, 25-vii-1971, Howarth (BPBM, 1 [male genitalia missing]), Weldon Sheldon entrance, downslope, 21-xi-2018, A. Engel/ Hudson/ Taylor, HI00713 (CUIC, 2), upslope, 21-xi-2018, C. Slay/ Yelverton/ Gunter/ Gracanin/ Hackell, HI00722 (UHIM, 2), 21-iii-2019, Chong, Hudson, A. Engel, S. Engel, Gracanin, lot HI01017 (UHIM, 1); Pahoa, Pahoa Cave, deep zone, 180 m el., 15-iii-1994, Howarth/ Miller (BPBM, 1), on bait, 18-iii-1994, Howarth/ Miller (BPBM, 1). **South Hilo District**: Kaumana Cave, 10-xi-2018, Chong, Hudson, Porter, Thomson, Lot HI00499 (UHIM, 1), 19-iii-2019, Engel/ Hackell/ Gracanin, Lot HI00925 (UHIM, 1), Porter/ Engel/ Hudson, Lot 00942, (CUIC, 1; UHIM, 2).

#### Diagnosis.

This is a small-bodied, pallid *Paratachys* with thin, translucent cuticle, and an iridescent sheen to the elytra due to the elongate transverse microsculpture; standardized body length 1.9–2.2 mm. The eyes are small but somewhat variable, with from 3–5 ommatidia crossed by a horizontal diameter of the eye, and from 4–6 ommatidia crossed by a vertical diameter (Fig. 9C–F); OR = 1.14–1.19. The elytra are broad, with broadly rounded humeri and broad, nearly truncate apices. The elytral apical recurrent groove is broadly, shallowly impressed. Sutural interneur 1 (i1) of each elytron is well impressed, with the two interneurs bracketing an elevated, callous-like elytral suture, whereas interneurs 2 and 3 are broadly, shallowly impressed to obsolete on the disc, with the outer interneurs absent except for the configuration of interneur 8 (i8) characteristic of *Paratachys* (Boyd and Erwin 2016: fig. 1A).

#### Description.

***Head*** narrow, ocular lobes nearly flattened anterad the genae in dorsal view (Fig. 9A); frontal grooves narrow mesad anterior supraorbital seta, broadest between anterior eye margins, and extended to lateral reaches of frontoclypeal suture; antennae moderately elongate, antennomere 9 length twice diameter; mandibles of moderate length, mandibles elongate, distance from dorsal condyle to apex 1.6 × distance from condyle to anterolateral labral margin; labrum transverse, anterior margin evenly emarginate across width, six-setose; penultimate maxillary palpomere broadly spindle-shaped, apical palpomere a narrow spindle (Fig. 9A). ***Prothorax*** variably transverse, MPW/PL = 1.33–1.46, with lateral margins distinctly sinuate anterad the acute, projected hind angles, MPW/BPW = 1.18–1.27, the sinuosity of the lateral margins accentuating the cordate appearance more than that represented by measuring pronotal width over the hind angles; pronotal median base depressed, its juncture with disc smooth, basal margin extended medially to form a collar that extends posterad the concave margins posterad the laterobasal depressions; laterobasal depression deepest as a lateral extension of the discal-median base juncture that is directed to the concave basal margin mesad hind angles; pronotal median impression finely depressed, narrow, pronotal disc flattened each side of midline; anterior transverse impression obsolete medially, broadly, shallowly extended laterally to narrowly rounded, moderately projected front angles. ***Elytra*** subquadrate, short, humeri broad, EL/MEW = 1.38–1.47; basal groove present laterad position of third interneur on interval distance laterad the parascutellar seta Ed1, groove convexly joined to lateral marginal depression; lateral marginal depression moderately reflexed, of equal breadth from seta Eo3 to subapical sinuation; broader Eo elytral setae elongate, e.g., seta Eo2 length = 0.7 mm, Eo9 length = 0.8 mm (Pahoa Cave, 15-iii-1994, ♂, BPBM). ***Pterothorax*** moderately elongate; mesepisternal depression smooth posterad juncture with mesosternum; metepisternum elongate, lateral length 1.6 × maximal width; metathoracic flight wings vestigial, the alae reduced to broad-based stenopterous flaps that extend nearly to posterior margin of first abdominal tergite (Fig. 9G) or to position of seta Eo4 when viewed through elytron, vestiges of radial, medial and cubital veins visible in the wing membrane. ***Abdomen*** with one seta each side of apical ventrite in males, two seta each side of ventrite in females. Microsculpture evident on all somites; frons covered with shallow transverse mesh, sculpticells isodiametric and cuticular surface rougher in broad frontal grooves; pronotal disc glossy with elongate transverse sculpticells producing silvery sheen, median base with distinct transverse lines medially, and irregular isodiametric sculpticells laterally; elytra iridescent due to mix of elongate transverse-mesh and transverse-line microsculpture, the transverse lines denser laterally; abdominal ventrites glossy laterally with shallow, swirling elongate-mesh microsculpture. ***Pelage*** present on head, prothorax, elytra, pterothorax, abdominal ventrites and legs; pelage on head, pronotal disc, and elytra comprising microsetae separated by distances subequal to setal length (Fig. 9C–F), the microsetae linearly arrayed along elytral interneurs as well as along ommatidial margins of the eyes (Fig. 9B); prosternum and mesosternum covered with sparse pelage medially; pelage of short microsetae present on abdomen in middle of ventrite 2 between metalegs, and progressively more broadly on ventrites 3–6 (absent dorsad arc of metaleg movement); anterior and posterior surface of prothoracic femora sparsely covered with microsetae, anterior (ventral) surfaces of meso- and metathoracic legs more densely covered with elongate microsetae, the setal bases situated more closely than microsetal lengths, trochanters and coxae similarly covered with microsetae. ***Coloration*** pale; vertex rufous to rufo-flavous, clypeus rufo-flavous, labrum flavous; antennomeres flavous, outer antennomeres 4–11 with slight smoky cast; maxillary and labial palps flavous; pronotum rufo-flavous; elytra rufo-flavous, elytral lateral marginal depression narrowly flavous medially; elytral epipleuron rufo-flavous, concolorous with thoracic and abdominal ventrites; legs flavous from trochanters outward; pro- and mesocoxae concolorous with outer leg segments, metacoxae rufo-flavous to match thoracic ventrites. ***Legs*** with only basal male protarsomere bearing a blunt, antero-apical process.

#### Variation.

A series of five individuals from Kaumana Cave, South Hilo District, include the largest individuals observed for this species. Their standardized body lengths range 2.1–2.2 mm, whereas all other individuals from caves in Hamakua, Puna, and Kau District range 1.9–2.1 mm standardized body length. The Kaumana specimens are also more heavily melanized, though their rufo-brunneous coloration is concolorous with the darkest individuals from the other localities. As neither of these attributes are diagnostic, and variation in male genitalia (below) does not support recognition of the Kaumana Cave individuals as representatives of a distinct species, all individuals described here are considered conspecific.

#### Male genitalia.

Aedeagal median lobe straight to slightly expanded near midlength, dorsal surface straight near midlength, evenly narrowed to rounded tip that is densely covered with sensilla (Fig. 3D–F); flagellar complex broadly hemi-ovoid, with parallel cuticular ridges emanating from densely sclerotized ventrobasal margin; narrow, strap-like right paramere with three setae on narrowly rounded apex; basally broad left paramere elongate, the parallel-sided apical portion with broadly rounded, three-setose apex. There is some variability observable among aedeagal dissections with the dissection of a beetle from Kazumura Cave (Fig. 3D) somewhat broader at midlength, and the aedeagus of a male from Kaumana Cave (Fig. 3F) more gracile than the male from Kau (Fig. 3E). The Kaumana male is larger than the other two (2.2 mm vs. 2.0 mm) consistent with the slightly longer aedeagus observed in that dissection (Fig. 3F vs. Fig. 3D, E). The position of the flagellar complex within the aedeagal shaft is to be discounted, as the entire internal sac is eversible, and its position in repose would vary depending on the level of saccal inversion.

#### Female reproductive tract.

Bursa copulatrix short, broad, with spermathecal duct entering near base of common oviduct (Fig. 10); spermathecal duct very long, by measurement of drawing with mapping opisometer, 1.7 mm (!); spermathecal reservoir indistinctly annulated, at right angle to basal atrium and duct, spermathecal gland duct entering spermatheca near bend; spermathecal gland with narrow duct leading to broader reservoir, the reservoir bearing a small apical assemblage of ductule-bearing secretory cells on a narrow duct; gonocoxa bipartite (Fig. 4C); basal gonocoxite 1 narrow, elongate, a single apical fringe seta near apex of lateral apodeme; apical gonocoxite 2 broadened basally, with lateral extension directed dorsad to ventral surface of coxite bearing two lateral ensiform setae; one dorsal ensiform seta just apicad position of apical lateral ensiform seta; two apical nematiform setae situated in elongate fossa situated halfway between position of dorsal ensiform seta and tightly rounded apex of gonocoxite 2.

#### Etymology.

The species name *Paratachys aaa* incorporates the Hawaiian word ‘*a‘a.‘ā*, meaning lava cave (Nā Puke Wehewehe ‘Ōlelo Hawai‘i 2020). Being a Hawaiian word, the epithet is to be treated as a noun in apposition.

#### Distribution and habitat.

*Paratachys aaa* has attained a very broad subterranean distribution that includes lava tube caves within Mauna Loa and Kilauea volcanic flows. The species prefers the deep zone of lava tube caves (Howarth 1982), and can thus be considered troglobitic. The type locality, Kazumura Cave northeast of Kilauea (Fig. 11), was formed approximately AD 1445 (Clague et al. 1999), during an eruption of Mauna Loa that lasted approximately 50 years. Pauoa Cave and Burn Cave near the eastern tip of Hawai‘i Island were formed from Puna volcanics derived from Kilauea eruptions that occurred less than 1000 years ago. Similarly, the caves in Kau accessed from sites in Ocean View Estates near South Point (Fig. 11) were all formed during eruptive episodes of Mauna Loa involving k3 flows that range in age from 750–1500 years old (Trusdell et al. 2005). The northwesterly Bobcat Cave north of the Mauna Loa caldera (Fig. 11) is situated at the margin of a k3 flow overlying an older k2 flow, giving an age of origin for that cave of at most 1500 years ago. And most recently, Kaumana Cave just WSW of Hilo, formed during the 1880–1881 eruption of Mauna Loa. Thus *P. aaa* occupies a disparate array of recently formed lava tube caves derived from several eruptive episodes. That these tiny beetles have attained such a broad distributional range among caves of different flows supports Howarth’s (1972) proposal that Hawaiian cave animals occupy both the larger lava tube caves accessible to humans (and collectors), but also the fractal network of mesocaverns ranging from 0.1–20 cm in diameter that connect those tubes. Add lignified organic layers sandwiched between older and newer flows (Moore et al. 1989) to the mix, and one obtains a complex web of interconnected subterranean voids that supports a variety of microorganisms and the food web built upon them. *Paratachys* beetles serve as an apex predator in this system (Howarth 1973), with their active foraging necessarily leading to dispersal among variously available and connected tubes and mesocaverns (Howarth 1983, 1987). Whereas above-ground elevational variation in habitats coupled with geographical variation in rainfall and temperature serve to define geographic distributions, the relatively more homogeneous subterranean voids would allow beetle dispersal among variously connected mesocaverns. Given that their bodies are only 2 mm long, requiring only occasional meals and encounters with conspecifics of the opposite sex to support persistent populations among the variously connected cave systems, *P. aaa* have dispersed underground to colonize a broad swath of recently built Hawai‘i Island.

## Discussion

At present we can account for five Hawaiian species of *Paratachys*, each representing a different major island. Given the relative ages of those islands, spanning Kauai to Hawai‘i Island (Carson and Clague 1995), we can postulate that the *Paratachys* lineage colonized Kauai, or perhaps an older now submerged island, and has undertaken progressive colonization of newer islands as they have become subaerial. Monophyly of the Hawaiian species is supported by the derived presence of the dorsal elytral seta Ed5–6 near the midpoint of the arc of the apical recurrent groove (Fig. 1A), not near its proximal apex as observed in all species from potential source areas of the Hawaiian lineage; those being Asia, the Palaearctic, North America, or even Africa (Andrewes 1925; Jeannel 1941, 1946; Lindroth 1966). Because all other reported *Paratachys* lack this potential synapomorphy, we cannot posit an outgroup of restricted character distributions or geographic distribution, limiting our ability to root the Hawaiian clade relative to other *Paratachys*. Nonetheless, a transformation series is evident among the male aedeagal configurations for the four species with described males (Fig. 3). Of those four, *P. terryli* differs in possession of an elongate flagellar complex (Fig. 3A). *Paratachys terryli* and *P. arcanicola* both exhibit an elongate median lobe, though *P. arcanicola* shares a more hemi-ovoid flagellar complex with the Maui and Hawai‘i Island species, *P. haleakalae* and *P. aaa*. Thus, there is a morphological transformation series that supports the biogeographic hypothesis of progressive colonization. Also, *P. terryli*, denoted as “*Paratachys* sp. ‘USA: Hawaii’”, was placed using molecular DNA data as the adelphotaxon to six terminal taxa that include European, North, Central, and South American species (Maddison et al. 2019). By these data, the lineage including Kauai’s *P. terryli* originated prior to diversification of the Holarctic and Neotropical *Paratachys* spp. included in that analysis. More extensive taxon sampling is required to test mutual monophyly of the Hawaiian vs. non-Hawaiian taxa, to determine the outgroup to the Hawaiian taxa, and to support estimation of the time of origin for Hawaiian *Paratachys*.

*Paratachys terryli* exhibits two infraspecific polymorphisms; ocular polymorphism with individuals presenting a more or less continuous range of eye sizes ranging from fully developed, i.e., macrophthalmic (Fig. 1C) to microphthalmic (Fig. 1I), and flight-wing dimorphism, with 18 of 19 specimens possessing vestigial wings (Fig. 1J), vs. one female being fully winged (Fig. 2A). Variation in eye development also occurs in the other Hawaiian *Paratachys*; e.g., *P. arcanicola* with individuals with eyes varying 5–7 ommatidia across, *P. haleakalae* (Fig. 8C, D), and *P. aaa* (Fig. 9C–F), though all specimens representing these species are micropterous. Analogous ocular variation has also been documented for two *Mecyclothorax* spp. of Hawai‘i Island, *M. rufipennis* Liebherr, and *M. konanus* Sharp (Liebherr 2008b: figs 176–180), but as in *P. arcanicola*, *P. haleakalae*, and *P. aaa*, all individuals of these species, indeed all Hawaiian *Mecyclothorax* (Liebherr 2015), are vestigially winged. It is only within *P. terryli* that both ocular polymorphism and flight-wing dimorphism has been demonstrated. If the flight-wing dimorphism of *P. terryli* follows the simple di-allelic model demonstrated for *Pterostichus anthracinus* Illiger wherein the macropterous allele is recessive (Lindroth 1946), whereas eye development exhibits many intermediate states (Fig. 1D–I) supporting polygenic inheritance, then these two polymorphisms must have independent genetic bases. It is also possible that individuals possessing the macropterous allele could have wing expression modified by environmental factors, such as larval nutrition, as shown for *Calathus melanocephalus* (L.) (Aukema 1990). Under such a hypothesis, macroptery and the development of flight muscles would be part of a developmental cascade governed by environmental factors acting at specific times on insect development, perhaps via modulation of endocrine controls (Harrison 1980). Perhaps eye development is also under control of such a developmental cascade? If so, larval experience and subsequent development could play an important part in governing adult outcomes.

Infraspecific ocular variation occurs among carabid beetle species across a broad taxonomic spectrum. *Melaenus elegans* Dejean (tribe Melaenini) sympatrically exhibits two eye morphs, microphthalmous and euphthalmous, toward the eastern parts of its range in Eritrea, Ethiopia, and Somalia, whereas only large-eyed euphthalmous individuals occur toward the western and southern extents of its African distribution (Ball and Shpeley 2005). Within tribe Zuphiini, the circum-Mediterranean *Parazuphium chevrolatii* (Castelnau) also exhibits dramatic variation in eye development (Assmann et al. 2015), with sympatric occurrence of large and small-eyed individuals within single populations. This species, like *P. terryli*, is also flight-wing dimorphic, with macropterous, macrophthalmic individuals observed in flight.

Elsewhere within Trechitae, populations of the western European-north African *Trechus fulvus* Dejean, a species ranging from Western Morocco, Spain, Madeira, and north to the British Isles and the Atlantic coast of Norway (Jeannel 1920; Lindroth 1985; Ortuño et al. 2017) exhibit both ocular and flight wing variation. Jeannel (1927) recognized six subspecies based on pronotal shape, eye size, male genitalic configuration, and body size, though recent treatments demonstrate that these subspecies do not adequately characterize the infraspecific variation (Faille et al. 2014). *Trechus fulvus primigenius*Jeannel (1920), recorded from Ciudad Real Province, Spain, and along the Rio Pomarâo, Portugal (Jeannel 1927), represents the most generalized populations found throughout the southern Iberian Peninsula (Ortuño et al. 2017); these are characterized by large eyes, darkly pigmented cuticle, and flight-wing polymorphism. Eye reduction has evolved independently among various cave-inhabiting populations of *T. fulvus* (Faille et al. 2014), consistent with the rampant parallel losses of eyes documented for cave-dwelling crayfish (Stern and Campbell 2018). The ocular polymorphism in *P. terryli* is perhaps more compelling, as the various ocular configurations occur among cohabiting beetles living in the same habitat. Based on the great variation in eye configuration among *P. terryli* beetles, selection for visual acuity has been relaxed (Stern and Crandall 2018), although a concomitant and opposing selective constraint for maintenance of a fully functional visual system in macropterous, flight-capable individuals is hypothesized.

A second dramatic finding regarding *Paratachys* beetles is the very broad, subterranean geographic distribution of *P. aaa* (Fig. 11). These cave beetles are distributed among lava tube caves derived from volcanic events involving both the Mauna Loa and Kilauea volcanoes. Moreover, these beetles are found in lava tube caves ranging in age from 3,000 years to only 150 years; i.e., Kaumana Cave. The beetles’ broad distribution dramatically affirms Howarth’s finding that underground dispersal into newly available Hawaiian lava tube caves happens incredibly rapidly (Howarth 1972, 1973, 1983, 1987). That this occurs among populations of beetles 2 mm long is remarkable, but if beetle populations are to persist in an area, then they must continuously colonize newly available, biologically productive caves. As predators, these beetles need access to lower-trophic level organisms which are ultimately dependent on plant roots and microbes growing with the benefit of moisture entering the cave from the surface. Given too much overburden from subsequent volcanic events, lava tube caves lose access to these resources and become sterile, as found by Borges et al. (2007) for caves in the Azores. Also, even though *P. aaa* can be considered troglobitic, as all records are from dark zones of lava tube caves, the adults retain small pigmented eyes and vestigial wings. Thus, they should be considered neo-endemic cave beetles, on a continuous rush to colonize newly available, maximally productive caves. These beetles must follow the dictum of the Red Queen: “Now, *here* you see, it takes all the running *you* can do to keep in the same place (Carroll 1871: 50).” This suggests that genetic divergence would be minimal among populations in the presently documented cave populations of *P. aaa*. The cave troglophile *Mecyclothorax aa* Liebherr also exhibits a broad geographic distribution including caves and voids within Hualalai and Mauna Loa flows (Liebherr 2008), suggesting that cave carabid beetles on Hawai‘i Island actively disperse to colonize newly available cave and mesocavern microhabitats that also support arthropod prey such as collembola, mites, and dipteran larvae (Howarth 1973; Bellinger and Christiansen 1974; Zacharda 1982).

The expansive geographic distribution of *P. aaa* stands in stark contrast to those of trechine cave carabid beetles of the Azores, where two species, *Trechus picoensis*Machado (1988) and *T. pereirai* Borges, Serrano and Amorim (2004), are distributed in caves of Pico Island; an island of 447 km^2^ surface area, less than 10% of the surface area of Mauna Loa volcano alone. The area of occupancy of *T. picoensis* is estimated at 40 km^2^, with the geographic range estimated to cover 285 km^2^, supporting categorization of this species as endangered (Borges and Amorim 2008). The more extensive subterranean distribution of *P. aaa* may lie partially in the greater physical dimensions of Hawaiian lava tube systems, with among others, Kazumura Cave of Kilauea Volcano, type locality for *P. aaa*, extending for more than 60 km while inclining more than 1100 m elevation. Windward Hawai‘i Island is also much wetter than the smaller Pico Island, thereby supporting ample forest tracts whose trees send roots deep into the volcanic soil, thereby entering the ceilings of lava tubes and smaller voids. Therefore, cave habitats suitable for carabid occupation may be more extensive and consistently productive in Hawai‘i.

Not all Hawaiian cave organisms are geographically widespread, with the highly restricted distributions of species in the herbivorous *Oliarus polyphemus* Fennah (Hemiptera: Cixiidae) cave planthopper complex (Wessel 2013) standing in stark contrast to the extensive distribution of the predatory *P. aaa*. Populations of the *O. polyphemus* complex have diverged so that six distinct species, definable by mitochondrial haplotype and male mating calls, occur among caves on Hualalai, Mauna Loa and Kilauea. *Oliarus* hoppers feed exclusively on roots of ohia lehua plants emerging from cave ceilings, and thus populations are tied to the presence of ohia trees living on the surfaces of flows above the caves. Dispersal among different cave systems is considered very rare and so it is hypothesized that populations are repeatedly established by very limited numbers of individuals, leading to founder-flush speciation. The six cave *Oliarus* species have speciated within subterranean lava tubes that formed during the past 200,000 years (Carson and Clague 1995), making them among the most rapidly speciating animals on Earth. However, even these planthoppers currently occupy caves no older than 10,000 years, indicating that they must also continuously colonize newly available lava tubes that provide the resources needed for their herbivorous way of life.

It is clear from this revision that the conservation status of Hawaiian *Paratachys* ranges from relatively stable to exceedingly tenuous, even terminal. *Paratachys arcanicola* was last collected by R.C.L. Perkins in the hills north of Honolulu, O‘ahu in the early 20^th^ Century. These forest habitats were home to a number of species absent from the collecting record since then, with those species presumably extinct (Liebherr 2009). And it is only fortuitous that Perkins collected a single specimen of Moloka‘i’s *P. perkinsi* between Kaunakakai and Kalua ‘aha in 1894. The species has not seen since and so its fate is unknown. It is only in the sodden summits of Kauai, the wettest rain forest of Kipahulu Valley, Maui, and the stygian voids of Hawai‘i Island that Hawaiian *Paratachys* beetles have been documented recently. Continuing survey of these habitats will enhance our knowledge of their geographic distribution, ecological status, and population structure, all necessary data for evaluating their conservation status.

## Supplementary Material

XML Treatment for Paratachys

XML Treatment for Paratachys terryli

XML Treatment for Paratachys arcanicola

XML Treatment for Paratachys perkinsi

XML Treatment for Paratachys haleakalae

XML Treatment for Paratachys aaa
